# Polymer Encapsulation of Bacterial Biosensors Enables Coculture with Mammalian Cells

**DOI:** 10.1021/acssynbio.1c00577

**Published:** 2022-03-04

**Authors:** Ignacio Moya-Ramírez, Pavlos Kotidis, Masue Marbiah, Juhyun Kim, Cleo Kontoravdi, Karen Polizzi

**Affiliations:** † Department of Chemical Engineering, Imperial College London, London SW7 2AZ, United Kingdom; ‡ Imperial College Centre for Synthetic Biology, Imperial College London, London SW7 2AZ, United Kingdom

**Keywords:** coculture, biosensor, bacteria, mammalian cells, l-lactate, hydrogel encapsulation

## Abstract

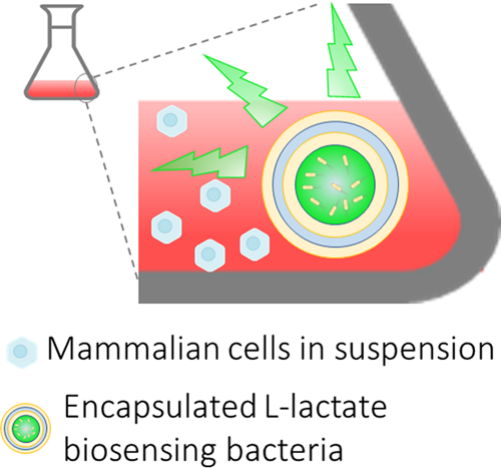

Coexistence of different populations of cells and isolation of tasks can provide enhanced robustness and adaptability or impart new functionalities to a culture. However, generating stable cocultures involving cells with vastly different growth rates can be challenging. To address this, we developed living analytics in a multilayer polymer shell (LAMPS), an encapsulation method that facilitates the coculture of mammalian and bacterial cells. We leverage LAMPS to preprogram a separation of tasks within the coculture: growth and therapeutic protein production by the mammalian cells and l-lactate biosensing by *Escherichia coli* encapsulated within LAMPS. LAMPS enable the formation of a synthetic bacterial–mammalian cell interaction that enables a living biosensor to be integrated into a biomanufacturing process. Our work serves as a proof-of-concept for further applications in bioprocessing since LAMPS combine the simplicity and flexibility of a bacterial biosensor with a viable method to prevent runaway growth that would disturb mammalian cell physiology.

## 1 Introduction

Like any other industrial process, bioindustries strongly rely on monitoring and controlling actions to maintain the optimal operating conditions.^1^ To that end, the concentrations of substrates and key metabolites need to be tightly monitored. This is commonly performed by electrochemical or chromatography techniques, which normally require destructive sampling and are used off-line.^2^ In this regard, there is a growing interest in harnessing the ability of living systems to interact with their environment to develop analytical devices, i.e., biosensors. They leverage the naturally evolved ability of organisms, cells, or biomolecules to detect and respond to a specific target molecule or ligand. Biosensors can compare favorably with their physicochemical counterparts in terms of sensitivity, specificity, and limit of detection.^3,4^ They are also capable of detecting complex analytes such as proteins in a simpler way compared to traditional approaches.^5,6^ Therefore, their versatility allows biosensors to find numerous applications for quantitative measurements,^7−9^ as diagnostic tools^10,11^ or as wearable devices.^12^ Furthermore, the use of standardized and modular parts and an engineering workflow that follows the design-build-test-learn cycle allows the implementation of more sophisticated designs to enhance the performance of the biosensor. Examples include the use of toggle or bistable switches,^13,14^ logic gates,^15^ and transcriptional amplifiers.^16^ Recent examples of more complex biosensor designs with higher functionality include the construction of oscillators to coordinate the dynamic behavior of thousands of colonies in response to the concentration of the analyte,^17^ the amplification of the biosensor response,^18^ or the integration of biosensors as a regulatory element to control the dynamics of the genes involved in the biosynthesis of chemicals.^19^ In addition, innovative designs are based on different kinds of output signals beyond the more traditional reporter proteins such as green fluorescent protein (GFP), including fluorescence resonance energy transfer (FRET) or structural changes in nucleic acids probes.^20−22^

Biosensors also possess an emerging potential to become a foundational technology for metabolite control in biomanufacturing. Here, whole-cell biosensors (based on living cells) are of particular interest because they can be easily programmed for control actions linked to biosensing, bringing new capabilities to a bioprocess. In addition, they are easy and inexpensive to produce once assembled. However, the integration of whole-cell bacterial biosensors in a biomanufacturing process will require strict compartmentalization to control the populations of the biosensing and producing cells to avoid undesired growth of the former and depletion of nutrients.

Here we establish a framework for the use of whole-cell biosensors in biomanufacturing. As a proof-of-concept, we have selected a bacterial–mammalian coculture, as it represents the biggest challenge in terms of differences in physiology and growth rate in a cell culture and remains underexplored for this kind of application. In particular, we employed an *Escherichia coli* whole-cell biosensor that expresses GFP in response to the concentration of l-lactate. This allows us to capitalize on synthetic biology tools widely established in *E. coli* for rapid biosensor development. However, because bacteria such as *E. coli* have high growth rates, they can easily overgrow the population of mammalian cells in the culture. Therefore, the main challenge is to balance the system so that both populations survive and function correctly. To overcome this challenge, we present a method to encapsulate the whole-cell bacterial biosensor, based on an inner hydrogel core carrying the *E. coli* biosensor coated by a polymeric multilayer shell to impart further physical and chemical stability that we have called living analytics in a multilayer polymer shell (LAMPS). We show how LAMPS produce a GFP signal in response to the l-lactate and coculture them with two different mammalian cell lines. Given the simplicity of the encapsulation described here, it would be possible to build different LAMPS modules to detect additional metabolites of interest or to produce different output signals to facilitate multiplexing. Therefore, LAMPS could be deployed widely in biomanufacturing processes by allowing self-regulated and preprogramed responses to key metabolites in a cell culture.

## 2 Results

### 2.1 Whole-Cell Biosensor Design and Optimization

**Figure 1:**
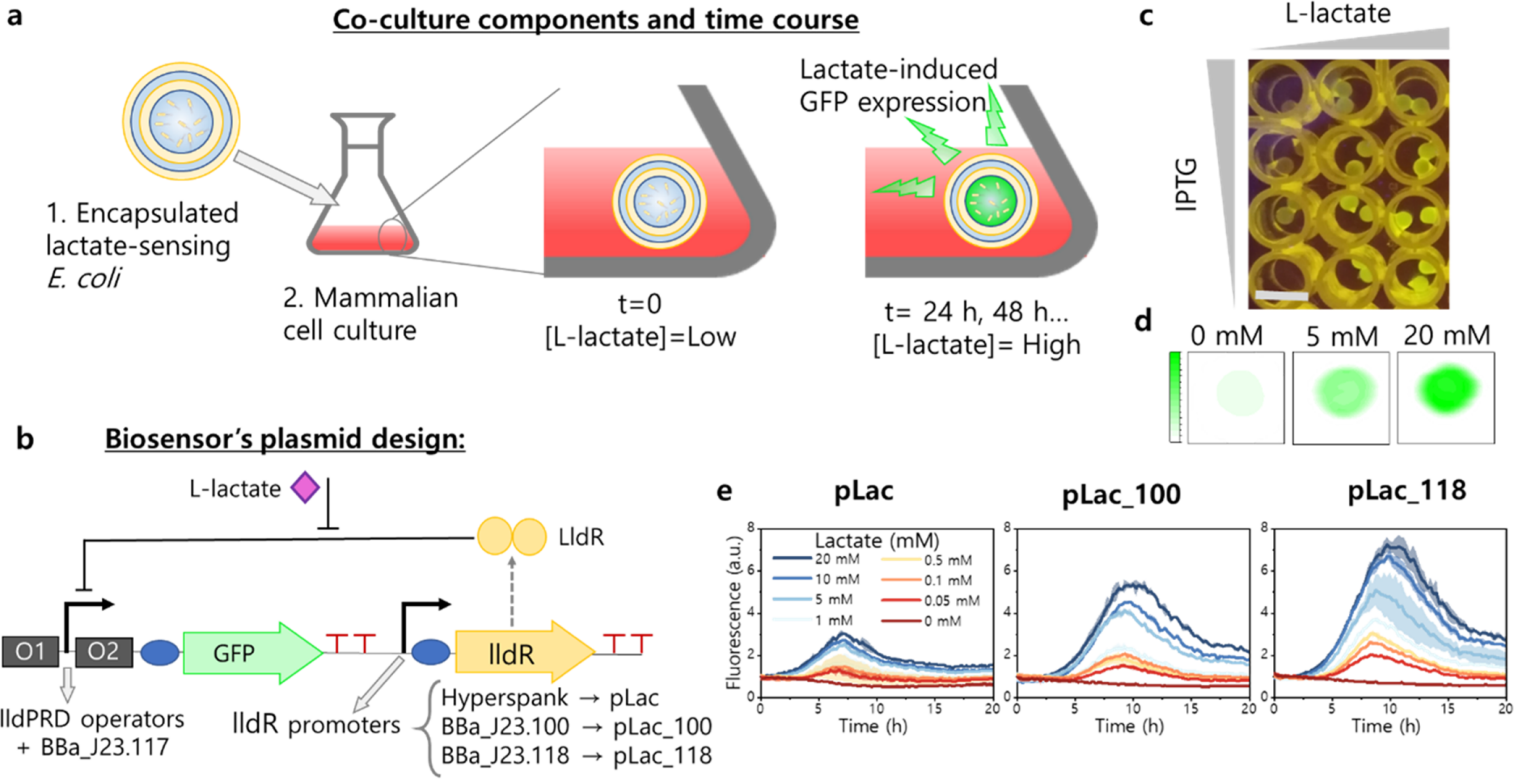
Coculture design and validation of the biosensor element. (a) Design of the bacteria–mammalian cell coculture proposed in this work comprising an *E. coli* whole-cell biosensor encapsulated in an alginate hydrogel matrix with additional polymer coatings and a free-growing mammalian cell culture. The containment of the bacteria in the hydrogel must ensure that no bacteria are released into the liquid medium and, at the same time, allow inward diffusion of the lactate. The increase in lactate concentration during mammalian cell growth induces the expression of GFP by the encapsulated lactate-biosensing *E. coli*, which remain inside the hydrogel during the course of the coculture. (b) Biosensor design for the detection of lactate. O1 and O2 represent the operator sites of the lldPRD promoter, flanking the constitutive promoter BBa_J23117. In the absence of lactate, LldR transcription factor binds to both operators. In the presence of lactate, LldR detaches from O2 and the expression of GFP is induced.^23^ Three different promoter upstream of the lldR gene were analyzed: hyperspank (IPTG-inducible), BBa_J23100, and BBa_J23118. (c) GFP fluorescence of *E. coli* cells carrying the pLac plasmid and encapsulated in an alginate hydrogel observed under blue light. Beads were incubated in buffer A, with 0-, 1-, or 10 mM lactate and 0.01, 0.1, 0.25, or 1 mM IPTG (to induce regulator expression) at 37 °C overnight. Scale bar: 6.8 mm. (d) Contour plots of the fluorescence scan across the surface of alginate beads incubated overnight in M9 with different concentrations of lactate. These fluorescence scans were used to analyze the GFP signal produced by LAMPS as a function of the concentration of lactate in the culture medium. (e) Lactate titration experiments with liquid cultures of *E. coli* carrying one of the three different lactate-sensing plasmids: pLac, pLac_100, or pLac_118. Cells were grown in M9 with glucose 0.4% as a carbon source. The lines represent the mean and shading indicates the standard deviation (*n* = 2). The equivalent experiment with glycerol is included in the Supporting Information (Figure S2).

We built a coculture consisting of an *E. coli* whole-cell l-lactate biosensor encapsulated in LAMPS and a free-growing mammalian cell culture (Figure 1a). The l-lactate responsiveness of LAMPS is encoded on a plasmid within the bacteria that carries an l-lactate-inducible promoter derived from the lldPRD operon in *E coli* and a transcriptional unit to overexpress the LldR regulator to reduce basal GFP expression (Figure 1b).^23,24^ We tested our initial biosensor plasmid, the β-d-1-thiogalactopyranoside (IPTG)-inducible pLac,^24^ in *E. coli* cells encapsulated in an alginate hydrogel (LAB). The LAB hydrogel cores prepared had a mean diameter of 1.6 mm (polydispersity index 0.00157, Figure S1). The increase in GFP fluorescence upon l-lactate addition confirmed that the *E. coli*-pLac cells retain their functionality when encapsulated, and therefore, suggesting that they can be used as the biosensing element in a coculture system (Figure 1c,d).

To simplify the workflow and eliminate the need for IPTG addition, we exchanged the IPTG-inducible Hyperspank promoter driving the expression of the LldR with a constitutive promoter. Two constitutive promoters of different strength, BBa_J23100 (strong) and BBa_J23118 (medium),^25^ from the Anderson library were cloned into the biosensor plasmid and tested with a l-lactate titration experiment in liquid culture. The original and both new designs show an l-lactate-dependent response within the commonly encountered range of l-lactate concentration in bioprocesses (0–20 mM) when tested in a medium containing glucose or glycerol as a carbon source (Figures 1e and S1). For both carbon sources, the design with the medium strength constitutive promoter pLac_118 had the highest signal-to-noise ratio (3.77, 8.41, and 10.34 for pLac, pLac_100, and pLac_118, respectively, after 10 h of incubation with 20 mM of l-lactate) while maintaining the same level of background signal and limit of detection (0.05 mM of l-lactate). A higher dynamic range is desired since it both increases sensitivity and results in a smaller number of encapsulated cells needed for the detection. Therefore, pLac_118 was selected as the best design available and used for all subsequent experiments.

### 2.2 Encapsulation of Biosensing Bacteria

#### 2.2.1 Optimization of LAMPS Coating

**Figure 2:**
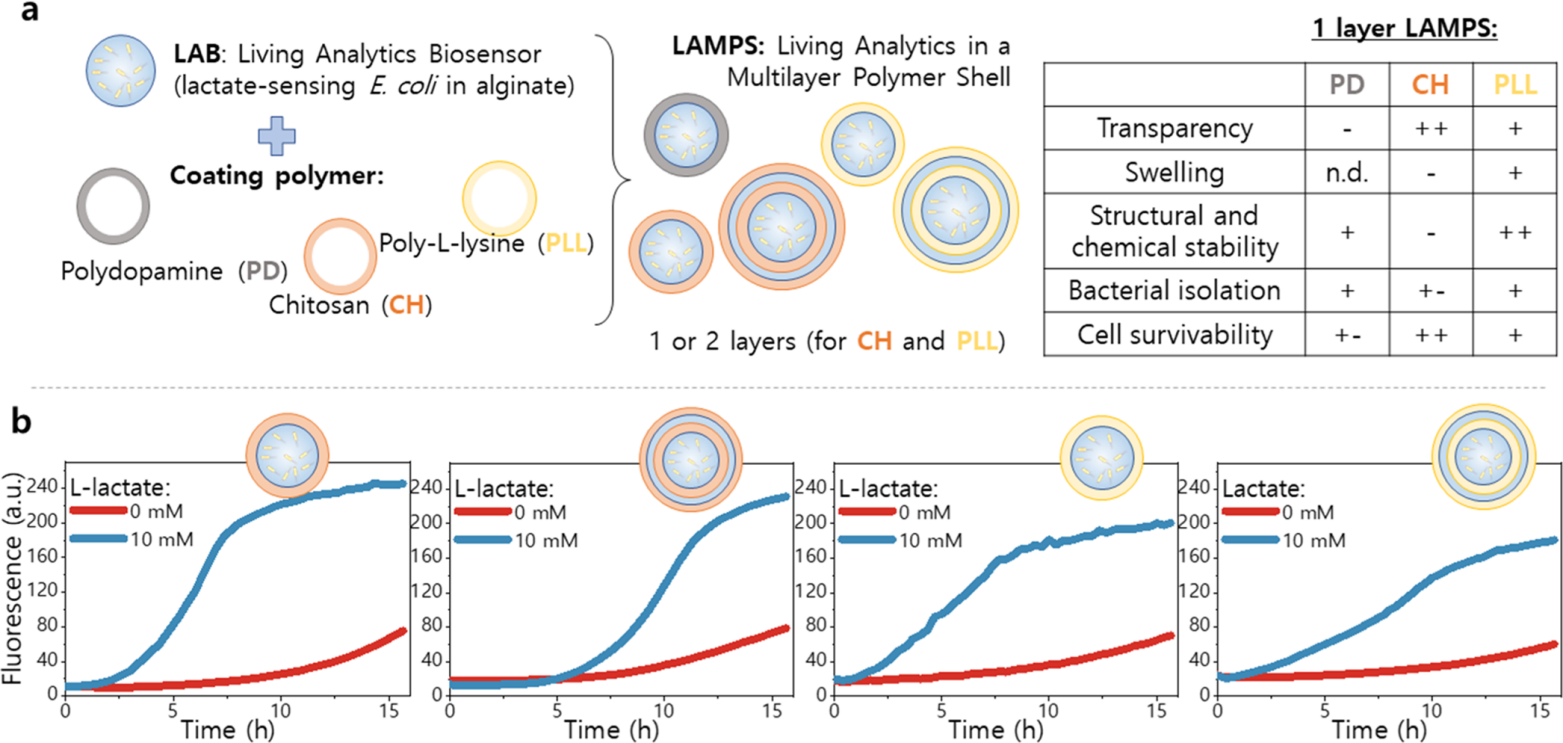
Optimization of LAMPS coating. (a) Elements of LAMPS: the LAB core, composed of an alginate matrix encapsulating lactate-sensing *E. coli* cells, is coated with a polymer of opposite charge to isolate the encapsulated bacteria and prevent their escape, creating LAMPS (living analytics in a multilayer polymer shell). Polydopamine (PD), chitosan (CH), and poly-l-lysine (PLL) were tested as coating polymers. The properties of LAMPS with one layer of coating polymer are summarized in the table (n.d. stands for not determined). (b) Fluorescence signal upon lactate induction in M9, for one- and two-layer LAMPS prepared with CH and PLL. From left to right: CH-1 layer, CH-2 layer, PLL-1 layer, and PLL-2 layer. For two-layered LAMPS, induction of the response was delayed around 2 h, but the maximum signal and background remained mostly unaltered.

Next, we studied how to contain the bacteria within LAMPS to avoid their escape and subsequent overgrowth/contamination of the mammalian cell culture. The containment strategy must allow sufficient diffusion of the analyte to preserve the biosensor responsiveness. Since alginate alone is insufficient to prevent bacterial escape, we decided to base our approach on a multilayer (onionlike) encapsulation, alternating layers of alginate (negatively charged), and positively charged polymers.^26,27^ In addition, the extra layers of coating can also compensate for the mechanical and chemical instabilities of alginate in the absence of divalent cations.^28^ While there is extensive research about coating hydrogel beads,^28^ few studies have examined the propensity for bacterial escape. Recent work has suggested that an outer coating consisting of photocrosslinked methacrylate,^29^ polydopamine (PD),^30^ or polyacrylamide^9^ help to prevent microbial release. Based on this, we selected polydopamine (PD), chitosan (CH), and poly-l-lysine (PLL) as positively charged polymers to explore (Figure 2a).

After coating with PD for 3.5 h, cells did not escape from LAMPS during an overnight incubation in buffer A, but colony-forming units were observed after plating samples from LAMPS that had been disrupted. However, no colony-forming units were observed on the plates when LAMPS were coated for 12 h with PD. This suggests that PD has some degree of toxicity for *E. coli*, even though it seems to be innocuous for *Saccharomyces cerevisiae*.^30^ In addition, PD coating is opaque, with an absorption spectrum covering the UV–visible range,^31^ which makes it incompatible with biosensing applications based on fluorescence excitation. Conversely, CH and PLL generated a translucent coating, with CH performing better in terms of cell survivability and coating transparency, while PLL conferred higher resistance to swelling (Figures 2a and S2). In addition, preliminary tests using LAMPS with one coating layer of either CH or PLL prevented the release of bacteria after incubation in buffer A in 3/4 and 4/4 samples, for CH and PLL, respectively.

The performance of LAMPS after the onionlike encapsulation was tested for both polycations (PCs), to ensure the desired containment of the bacteria in a culture environment. LAMPS with one (LAB-PC) and two (LAB-PC-alginate-PC) coating layers were incubated in M9 culture medium in a 96-well plate, and the fluorescence was monitored every 20 min. Figure 2b shows the response of LAMPS with one and two PC layers in the presence and absence of l-lactate. LAMPS prepared with both CH and PLL showed a clear response when the media was spiked with l-lactate (10 mM), confirming that it is possible to perform monitoring of the fluorescence signal of the living biosensor without the need to disrupt the LAMPS first. In the absence of l-lactate, a stable background response was recorded during the first 10 h of incubation.

Overall, the number of layers had a significant effect on the dynamics of the LAMPS response, particularly on the lag phase. The activation of the LAMPS with one layer of positively charged polymer was around 2 h faster in culture medium compared to those with two layers, for both CH and PLL, though the opposite occurred when LAMPS were incubated in buffer A, which lacks a carbon source (Figure S4). This suggests that the diffusion of nutrients and/or l-lactate is slower in the multilayer coating, probably due to the thicker coating layer. However, for both positively charged polymers and regardless of the number of layers, the maximum signal and background level were similar at the end of the incubation.

We also tested the performance of CH and PLL coatings in bigger culture volumes, incubating LAMPS overnight in 1 mL of M9 in a 24-well plate, instead of in 200 μL as in the previous experiment. Here, the CH-coated LAMPS cracked open, exposing the LAB core (Figure S3b). Therefore, since PLL coatings prevented cell escape, LAMPS swelling and maintained their integrity in bigger volumes; these were used for all subsequent experiments.

#### 2.2.2 Dose–Response, Preservation, and Soiling of LAMPS

**Figure 3:**
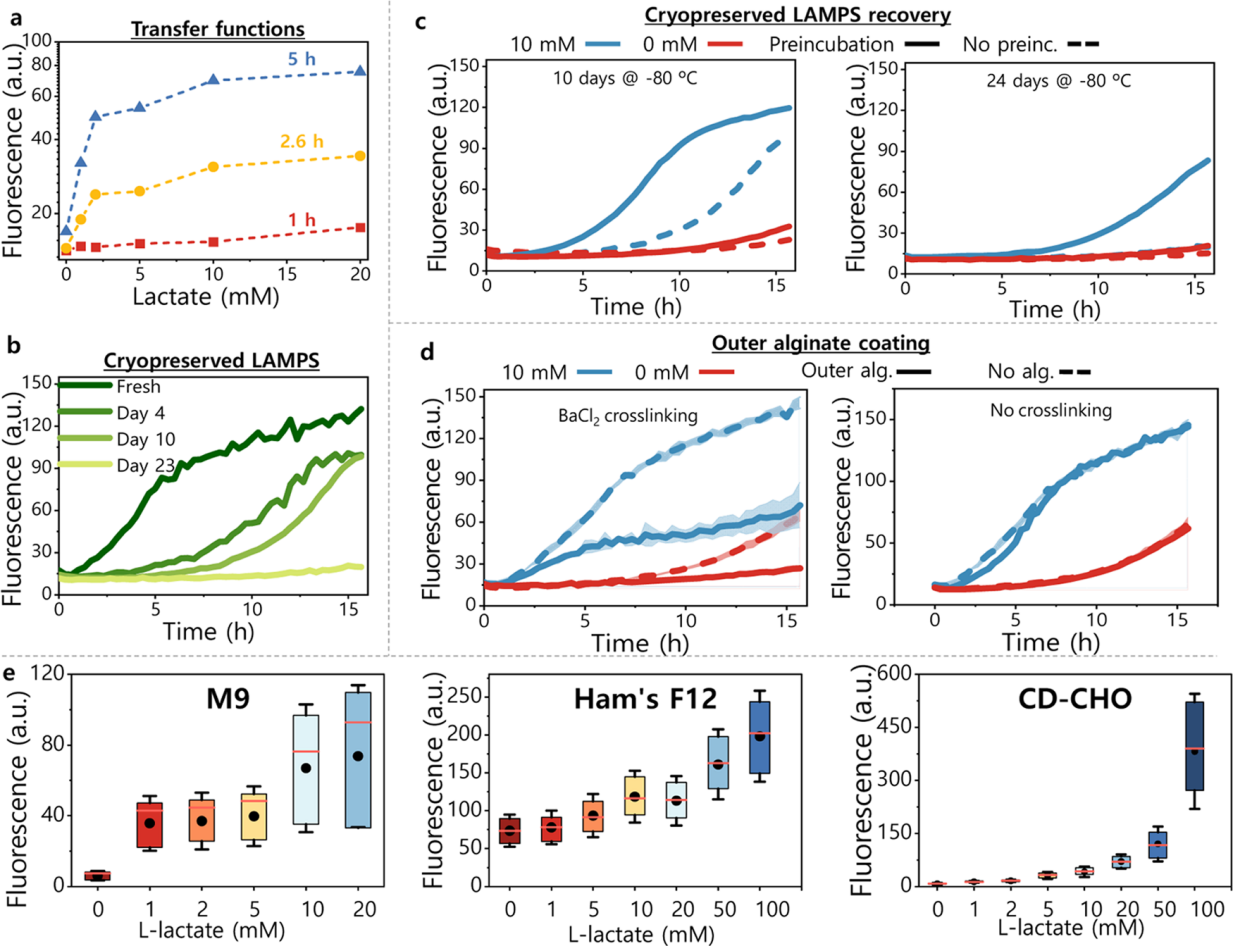
LAMPS dose–response, cryopreservation, and soiling reduction. (a) Transfer functions of freshly prepared LAMPS as a function of the lactate concentration after 1, 2.6, and 5 h of incubation. (b) Effect of cryopreservation on LAMPS response. Time course of the response of LAMPS at 20 mM of lactate after several days of cryopreservation. (c) Effect of preincubation in growth medium on the LAMPS response, for beads frozen 10 and 24 days (left and right, respectively). LAMPS were preincubated in M9 for 1 h before beginning the lactate induction experiment where 10 mM (blue) and 0 mM (red) of lactate were tested. Straight lines: preincubated LAMPS; dashed lines: no preincubation. (d) Effect of an additional outer layer of alginate crosslinked with BaCl_2_. For both cases, alginate-coated LAMPS (straight lines) were compared with those finished in a PLL outer layer (dashed lines). Responses at 0 and 10 mM of lactate (red and blue lines, respectively) were tested. All experiments shown in this figure were carried out in a 96-well plate, with one LAMP bead per well and 200 μL of M9. The plots display the average fluorescence values of the scanned surface in the plate reader at each time point. The lines represent the mean and shading indicates the standard deviation (*n* = 2). (e) Fluorescence signal of LAMPS as a function of the concentration of l-lactate, incubated in 1 mL of different media. The fluorescence reads obtained by the scan of the surface of LAMPS are presented as a box chart. The box contains the reads within the percentiles 25–75%; the error bars represent the mean of all of the values ± standard deviation (SD); the mean is indicated by a circle and the median by a horizontal red line.

We next studied some features of LAMPS that are relevant for their performance as a biosensor in a coculture. First, we tested the response of LAMPS to a range of metabolically relevant concentrations of l-lactate and calculated the transfer functions after 1, 2.6, and 5 h of incubation (Figure 3a). The increase in fluorescence with the increase in l-lactate and over time confirms that LAMPS are responsive to l-lactate concentrations up to at least 20 mM. However, at longer times, the signal saturated at concentrations greater than 2 mM in small volume cultures (Figure S5). Interestingly, when LAMPS were cultured in larger volumes, the range of detection was extended up to 20 mM (see the end of the section and Figure 3e).

We also tested the cryopreservation of LAMPS as a solution to tackle batch-to-batch variability and the need to prepare them just before use. Figure 3b compares the response of freshly prepared LAMPS and those cryopreserved at −80 °C for several days. The maximum signal decreased as the storage time increased. Nonetheless, LAMPS remained responsive to l-lactate and the *E. coli* cells did not escape during the experiment, suggesting that the integrity of the polymer shell is not affected by freezing or storage. Since the decrease in the signal can stem from a reduction of the number of viable *E. coli* inside LAMPS,^9^ we attempted to recover the bacterial population by preincubation in a fresh medium to reactivate the cells prior to repeating the l-lactate titration experiment. With reactivation, LAMPS frozen for 10 days had an l-lactate response similar to freshly prepared LAMPS (Figure 3c). Furthermore, LAMPS cryopreserved for 24 days retained ∼50% of the maximum signal when reactivated before use compared to no signal at all without reactivation (Figure 3c).

Next, in order to improve the measurement of the fluorescence signal, we aimed to reduce surface soiling by adding an additional outer layer of alginate (Figure S6). Given the reversible nature of alginate crosslinking with Ca^2+^ ions, Ba^2+^, a stronger crosslinker, was used to achieve a more stable outer layer.^32^Figure 3d shows that the addition of a Ba^2+^-crosslinked outer alginate layer (LAB-PLL-Alg-PLL-Alg_Ba_) considerably reduces the fluorescence signal compared to LAMPS without it (LAB-PLL-Alg-PLL). However, the outer layer visibly reduced the soiling of the beads after an overnight incubation in several different media (Figure S6). Moreover, the background signal of LAMPS terminating with alginate remained almost unchanged over a 16 h incubation, as opposed to the signal drift from 10 h onwards for those without it (Figure 3d). Taking into account the lower background, the reduction of the fluorescence signal from the additional layer is less relevant. The data also suggest that the surface soiling may interfere with fluorescence detection. When the Ba^2+^ was omitted, and therefore the alginate outer layer was not crosslinked, the fluorescence was similar to LAMPS with a PLL outer layer (Figure 3d) and only a minimal reduction in soiling was observed (Figure S6), suggesting that the crosslinking is necessary to retain the final alginate outer layer.

Taking this as the final design, we compared the response of LAMPS incubated in 1 mL of two types of fresh mammalian cell culture media (CD-CHO and Ham’s F-12) supplemented with l-lactate (Figure 3e, center and right). In each case, the signal proportionally increased with the l-lactate concentration between 0 and 100 mM. The differential response compared to the experiments in Figure 3a conducted in 200 μL highlights the importance of the medium/LAMPS volume ratio as a parameter affecting the signal of the biosensor, which may require optimization for future use.

### 2.3 LAMPS as Biosensing Elements in Mammalian–Bacteria Cocultures

Finally, we tested the ability of LAMPS to act as l-lactate biosensing units in coculture with mammalian cells. For this, we grew two Chinese Hamster Ovary (CHO) cell lines: adherent Flp-In CHO cells or IgG-producing CHO (IgG-C) suspension-adapted cells as cocultures with LAMPS.

**Figure 4:**
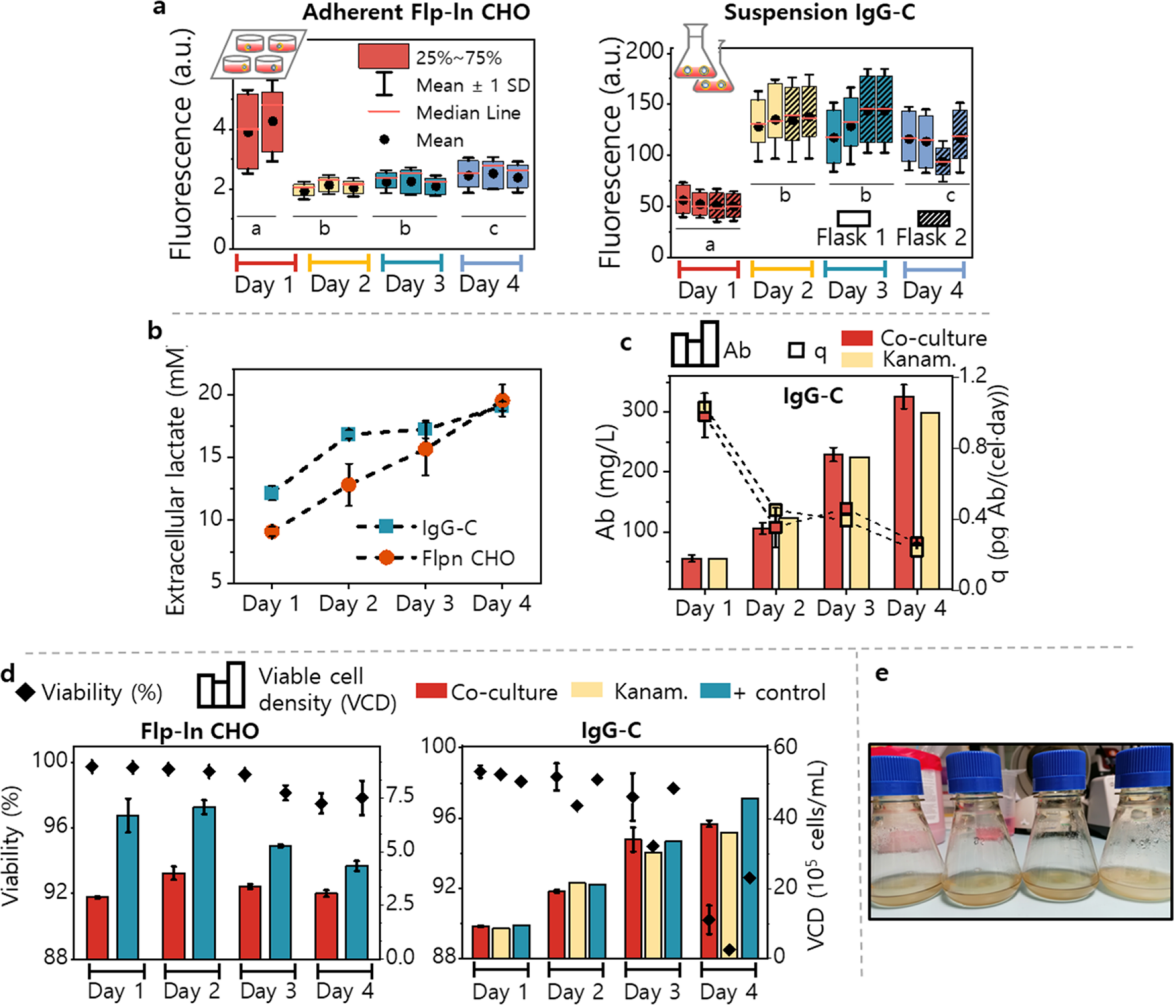
Cocultures of mammalian cells and LAMPS. (a) Box plots of the fluorescence reads from LAMPS after 20 h of coculture with mammalian cells. Each box presents the fluorescence reads obtained by the scan of the surface of one LAMPS. The box contains the reads within the 25–75% percentile; the error bars represent the mean of all of the values ± SD, where the mean is indicated by a circle and the median by a horizontal red line. For adherent Flp-In CHO (left), each experiment was conducted in duplicate or triplicate, while for suspension IgG-C cells (right), experiments were conducted in duplicate with two LAMPS in each flask. The letters (a–c) inset in the graphs indicate significant differences in the means of the fluorescence reads for each day according to a Tukey test (*p* < 0.01). (b) Extracellular lactate concentration in the coculture supernatants measured by an enzymatic assay. (c) Titer in mg/L (bars) and specific productivity in μg/10^5^ cell/day (squares) for the antibody produced by the IgG-C cells during the coculture experiments. (d) Culture viability (diamonds) and cell density (bars) of the mammalian cells. For (b)–(d), error bars indicate the SD of the mean values of the biological replicates shown in (a). (e) Images of flasks on day 4 of the experiment with IgG-C cells after 20 h of incubation. From left to right: cocultures with two LAMPS added (duplicates), kanamycin control and negative control using LAB cores (beads where the biosensing *E. coli* are encapsulated but not covered with successive PLL and alginate layers).

The two cell lines were grown in batch culture with no feed addition for 1–4 days. LAMPS were added to each culture at different times to measure the l-lactate concentration. After 20 h of coculture, LAMPS were recovered to perform the fluorescence readings (Figure 4a). For adherent Flp-In CHO, each experiment was conducted in duplicate or triplicate, in wells with one LAMPS bead in each well. For suspension IgG-C cells, experiments were conducted in duplicate with two LAMPS in each flask, giving a total of four LAMPS scanned for each day.

Fluorescence readings from the adherent Flp-In CHO cells were very low across the whole experiment and lower than that of the LAMPS incubated with culture medium spiked with l-lactate (Figure 3e). This suggests either the depletion of a key nutrient enabling bacterial biosensor functionality or the production of a metabolite that quenches fluorescence after the Flp-In CHO cells has been growing in the culture medium. In contrast, for cocultures of suspension IgG-C cells, the fluorescence was the lowest on day 1, increased on day 2, and showed a slight decrease on day 4. In this case, the fluorescence of LAMPS matches the trend of extracellular l-lactate concentration (Figure 4b).

In addition to LAMPS fluorescence, we also measured the cell density, viability, and, in the case of the IgG-C cells, monoclonal antibody production of the cocultures and compared these with the monoculture controls. Remarkably, the titer and antibody productivity of the IgG-C cells remained unaltered in cocultures with LAMPS (Figure 4c). In addition, the bacteria did not have a significant effect on the cell viability or density of either cell line when compared to positive control CHO cells grown alone (Figure 4d). Finally, for both cell lines and for all biological replicates, no release of *E. coli* was detected after 20 h of coculture, confirming that LAMPS provided total containment of the encapsulated bacteria in an actual coculture environment (Figure 4e). For comparison purposes, a negative control experiment with LAMPS lacking coating layers (LAB with a single layer of alginate only) was performed. Here, the proliferation of the released *E. coli* was clearly observed after the 20 h incubation (Figure 4e, far right).

## 3 Discussion

The interest in coculture spans from the study of cell–cell interactions to the improvement of production processes.^33−36^ Microbial communities have been extensively used to distribute labor and reduce metabolic burden,^37^ making use of natural or synthetic interactions.^38,39^ In the particular case of mammalian–microbe cocultures, they are of interest as models for infection studies, for example, in microbiota–host interactions.^40−44^ However, their application to manufacturing is less explored, even though cocultures represent a point of focus for the expansion of biomanufacturing.^45^ Some early examples that have been recently demonstrated are cocultures of epithelial cells and *Pichia pastoris* or HEK293T cells and engineered auxotrophic *E. coli*.^46−48^ However, applications of interkingdom cocultures for manufacturing have not been reported to date, in part due to the existing gap in compartmentalization technologies that allow both types of cells to survive and function correctly. This work demonstrates vital progress in this direction by applying a rationally designed and optimized biosensor of a key metabolite in biomanufacturing paired with a simple encapsulation methodology to permit the coculture of l-lactate-biosensing bacteria with mammalian cells. This allows a division of labor where the two types of cells share the same culture environment, but each maintains its functionality. The methodology is modular and readily extensible to other types of biosensors, cell types, and applications, making cocultures broadly useful for the implementation of synthetic biology tools such as biosensors or regulatory networks into biomanufacturing.

Among the various options to contain bacteria, we opted for hydrogel encapsulation due to its simplicity, flexibility, and the wide range of available materials.^49^ Alginate is the most commonly used matrix for encapsulation because of its ease of handling and biocompatibility.^50^ However, the main challenge for the construction of LAMPS is the need for full containment of the encapsulated *E. coli* to prevent contamination. We show that a multilayer coating of alternate layers of alginate hydrogel and CH or PLL completely prevents the escape of the encapsulated bacteria while maintaining their ability to sense and report the concentration of l-lactate in small volumes of M9 medium. The delay in the LAMPS response when adding successive CH or PLL layers suggests a decrease in the diffusion rate of l-lactate and/or nutrients to the interior of LAMPS and confirms the stacking of polycations and alginate layers onto the LAB core. In contrast, Mao et al.^27^ reported no decrease in the rate of diffusion of dextran molecules up to 200 kDa to the interior of alginate-PL-alginate micrometric beads. This suggests that factors other than the molecular weight, such as size, charge, or electrostatic attraction/repulsion, might have an effect on the diffusion of molecules in and out of the beads and should be considered for each particular application. Further experimentation showed that CH coating failed when testing LAMPS in culture volumes bigger than 200 μL. This could be due to increased diffusion of Ca^2+^ ions out of the alginate gel core or because of lower physical resistance. The effect of Ca^2+^ diffusion can be tackled by supplementing the incubation medium with Ca^2+^. However, this may be undesirable when mammalian culture media are involved, since they have well-defined and complex formulations. Therefore, since CH coating was unable to maintain structural integrity, we chose PLL as the best coating counterion for the preparation of LAMPS. We also found that LAMPS with an outer alginate layer has reduced soiling, particularly when crosslinked with BaCl_2_, leading to a lower background signal and preventing signal drift.

Similar to the behavior of the liquid cultures of the unencapsulated whole-cell biosensor, LAMPS showed a dose-dependent response when exposed to a range of l-lactate concentrations within normal metabolic levels, both in bacterial growth medium (M9) and in two types of mammalian cell culture media (Ham’s F-12 and CD-CHO). We observed that the medium itself had an important effect on the induced and background signals of LAMPS. For example, LAMPS culture in Ham’s F-12 had a background fluorescence around 10 times higher than that in M9 or CD-CHO. This could be attributed to differences in the growth of *E. coli* in different media with varying nutrient sources or variations in the concentration of other autoflorescent species.

The results also demonstrate successful cryopreservation of LAMPS, which can contribute to the production of standardized biosensors that provide reliable and reproducible measurements. We found that incubation in a fresh medium to recover the bacterial population allowed the use of cryopreserved LAMPS that had been stored up to 10 days with responses similar to freshly prepared ones. In future, the cryopreservation storage limit might be further extended with the use of cryoprotective substances and/or lower cryopreservation temperatures.^27,51^

Finally, we used the optimized LAMPS encapsulation to combine a living biosensor with a mammalian cell culture. LAMPS produced a GFP signal during their coculture with IgG-C but appeared to be quenched when cocultured with Flp-In CHO. This suggests that the signal is influenced by factors other than just the concentration of l-lactate. In particular, the consumption of nutrients by the mammalian cells during the monoculture period may reduce *E. coli* growth rate, which can explain the drop in the signal of LAMPS after day 3 for IgG-C. This multivariate effect makes it challenging to calibrate LAMPS fluorescence versus the concentration of l-lactate to enable absolute quantification since the calibration curve would need to be adapted to each medium, culture stage, and cell line (and may require a calibration curve in spent medium at each time point for a high degree of accuracy). Therefore, more research would be necessary to adapt LAMPS to other cell lines and media and to enable accurate quantification of l-lactate. Nonetheless, these results show for the first time an approach to perform mammalian–bacteria cocultures, where both kinds of cells can coexist in a controlled fashion and perform separated tasks. Remarkably, for both mammalian cell lines, there were no significant differences in culture viability and density between the cocultures and the monoculture positive control. More importantly, this was also the case for the productivity and titer of a recombinant antibody produced by the IgG-C cells. This confirms that the energy requirements of the *E. coli* encapsulated in LAMPS did not impose a detectable burden on the mammalian cells. The proof-of-concept demonstration shown here enables the application of other whole-cell biosensors in cocultures, addressing compatibility issues that restrict the practical use of many synthetic microbial biosensors.^52^ Overall, our results demonstrate that LAMPS can be used in the rational design-build-test-learn cycles in synthetic biology because of their modularity and ease of preparation, which can seed new applications of engineered cells as tools in biomanufacturing.

## 4 Conclusions

In this work, we have shown that LAMPS enable the construction of a synthetic bacterial–mammalian cell coculture with a predesigned separation of tasks. This represents a demonstration of a living biosensor that can be integrated into a biomanufacturing process. We envisage that our work provides a new methodology for the analysis of cells during production that can facilitate bioprocess development to increase yields and product quality. LAMPS may be very useful for processes run in systems such as wave bags or miniature bioreactors, where fewer analytical probes are available. The concept could be expanded to include arrays of biosensors for different molecules, given the flexibility for their design.^53^ Capitalizing on the modularity and simplicity of LAMPS, it would be easy to spatially arrange different engineered biological tools in a coculture or even to build circuits that combine different LAMPS modules, in line with the “plug and play” view of synthetic biology.^33^ Furthermore, LAMPS biosensing could be easily repurposed to distributed and modular control elements. The replacement of the GFP fluorescence signal with an active molecule (secreted enzyme or transcription factor, for example) will make it possible to couple the detection of metabolites with an action affecting the liquid culture or to interface with an electronic system.^54^ LAMPS can expand the applicability of the wide array of engineered cells made available by synthetic biology research to practical use in biomanufacturing. In particular, LAMPS could be used as living control units, enabling an autonomous and dynamic regulation of l-lactate concentration or other relevant parameters.

## 5 Methods

Salts and other ingredients for buffers and bacteria culture media, dopamine hydrochloride, fetal bovine serum (FBS), l-glutamine, sodium l-(+)-lactate, kanamycin sulfate, β-d-1-thiogalactopyranoside (IPTG), and phosphate-buffered saline (PBS) were obtained from Sigma-Aldrich (St. Louis, MO). Alginic acid sodium salt from brown algae (ref 71238), poly-l-lysine hydrochloride (MW 15 000–30 000 Da), and low-molecular-weight chitosan (ref 448869) were also supplied by Sigma-Aldrich. Chemically competent *E. coli* NEB5α cells were purchased from New England Biolabs (NEB, MA).

M9 culture medium was prepared with 33.7 mM Na_2_HPO_4_, 22 mM KH_2_PO_4_, 8.55 mM NaCl, and 9.35 mM NH_4_Cl as 10× stock and autoclaved. It was supplemented with 0.4% d-glucose (or glycerol), 1 mM MgSO_4_, 0.3 mM CaCl_2_, and 1 mg/L thiamine from stocks that were prepared separately and filter-sterilized. Krebs–Ringer *N*-(2-hydroxyethyl)piperazine-*N*′-ethanesulfonic acid (HEPES) (KRH buffer) buffer was prepared with 20 mM HEPES, 135 mM NaCl, 5 mM KCl, 0.4 mM K_2_HPO_4_, adjusted to pH 7.4, autoclaved, and supplemented with 1 mM MgSO_4_, and 1 mM CaCl_2_ from stocks prepared separately and filter-sterilized. Incubation buffer (buffer A) consisted of 10 mM HEPES, 150 mM NaCl, 20 mM CaCl_2_, and disruption buffer (buffer B) of 0.1 M ethylenediaminetetraacetic acid (EDTA) and 0.2 M potassium citrate; both were filter-sterilized.

### 5.1 Whole-Cell Biosensor Preparation and Encapsulation

The biosensor used in this work (Figure 1b) was adapted from Trantidou et al.^24^ by replacing the inducible Hyperspank promoter K143015, controlling the expression of the LldR transcription factor, with the promoters J23100 or J23118 (see Supporting Methods for sequences).

*E. coli* cells from precultures, grown as described in the Supporting Methods, were pelleted at 7000 g for 5 min and resuspended in fresh M9 to OD_600_ of 2. Solutions of 2% sodium alginate and 100 mM CaCl_2_ were prepared in 10 mM Tris pH 8.5, as described in Kim et al. and filtered-sterilized with a 0.45 μm syringe filter.^30^ The *E. coli* suspension and the alginate solution were thoroughly mixed in a 1:3 volume ratio (0.25 and 0.75 mL, respectively) in a sterile Eppendorf tube, and the mixture was drawn into a 1 mL syringe. Alginate–bacteria beads were formed by dropwise addition of the mixture from a height of 1 cm into 100 mL of CaCl_2_ solution in a sterile glass bottle under gentle magnetic agitation using a sterile 30G-blunt end needle (RS Components, U.K.). The alginate hydrogel beads were crosslinked for 30 min, collected with a sterile cell strainer (Fisher, U.K.), washed with fresh sterile CaCl_2_ solution, and incubated for 1 min in 10 mL of KRH buffer in a Petri dish. The resulting beads after this step are labeled as living analytic biosensors (LABs) in the manuscript.

For the addition of polymer coatings to create LAMPS, LABs were covered with successive layers of a polymer by dip coating. Here, LAB was transferred to a 15 mL Falcon tube containing 5 mL of a solution of 1 mg/mL poly-l-lysine (PLL) in KRH buffer, incubated for 10 min under gentle agitation and washed with fresh KRH buffer. When desired, the second layer of alginate was added by the subsequent incubation of the beads in a 0.2% alginate solution in 10 mM Tris buffer pH 8.5 for 10 min. Beads were washed with KRH for 1 min and again incubated in the PLL solution for 10 min. The PLL solution was removed and the beads were washed with 5 mL of sterile in 0.15 M mannitol. Next, 5 mL of 1 mg/mL PLL in 0.15 M mannitol was added, incubating the beads for 2 h. Finally, the effect of an additional outer layer of alginate was also studied. For that, the beads were rinsed with KRH buffer, incubated in a 0.2% alginate solution in 10 mM Tris buffer pH 8.5 for 10 min, washed with KRH, and finally crosslinked in a solution of 50 mM BaCl_2_, 0.15 M mannitol for 5 min. After the addition of the last coating layer, LAMPS were rinsed with KRH buffer. Beads not used directly after preparation were cryopreserved in a 1:1 mix of incubation buffer and glycerol 50% in 1.5 mL Eppendorf tubes. They were initially frozen at −18 °C and moved to −80 °C after 24 h. When desired, beads were disrupted by incubation in 1 mL of buffer B at 37 °C for at least 10 min. Modifications of the described protocol for coatings with dopamine, and chitosan are detailed in the Supporting Information.

### 5.2 Coculture Experiments

Two CHO cell lines were used. Suspension-adapted Chinese hamster ovary cells producing an IgG antibody (IgG-C) were maintained in CD-CHO medium (Life Technologies, Paisley, U.K.) at 36.5 °C, 150 rpm, and 5% CO_2_. Cells were passaged three times every 3–4 days prior to the coculture experiments, at a seeding density of 3 × 10^5^ cell/mL. The second cell line was the adherent Flp-In CHO (Thermo Scientific, EEUU). These were seeded at a 1:10 density in T75 flasks with an adherent surface and vented cap (Sarstedt). Cells were grown in Ham’s F12 (Sigma) containing 10% FBS and 2 mM l-glutamine (according to manufacturer’s instructions). Flp-In CHO cells were grown at 37 °C with a 5% CO_2_ atmosphere and also subcultured three times prior the coculture experiment.

Coculture experiments were conducted over 4 days to test the response of LAMPS in different growth phases using cryopreserved beads from the same batch for each time-course experiment. LAMPS were prepared 6 days prior to the experiment and frozen and stored at −80 °C as described above. Each day prior to their use, LAMPS were defrosted at 4 °C for 1 h, washed with fresh M9, and incubated in M9-glucose at 37 °C for 1 h (12 beads/5 mL of medium). LAMPS were rinsed with PBS and individually transferred to the mammalian cell culture with a sterile plastic Pasteur pipette. LAMPS beads were cocultured for 20 h with CHO cell cultures previously grown for 1–4 days, under the same conditions as those used for mammalian cell monoculture. In the coculture experiments with adherent CHO cells, one LAMPS bead was added to each well of a six-well plate along with 2 mL of cell culture. For suspension cells, two LAMPS were added to 20 mL of IgG-C cell cultures in 125 mL Erlenmeyer flasks. Both experiments had at least one independent duplicate. Kanamycin was supplemented to a concentration of 37.5 mg/L at the beginning of the coculture. Positive controls consisting of mammalian cell cultures in the absence of LAMPS were used to assess cell growth and productivity, with and without the addition of kanamycin. Negative controls using LAB (without the PLL and alginate coating layers) were also used.

The Supporting Information contains additional information about methods used to measure culture viability and cell density, IgG antibody and l-lactate concentration, and fluorescence measurements in LAMPS.

### 5.3 LAMPS Fluorescence

For the fluorescence measurements, LAMPS were placed in 96-well spheroid microplates (Corning). The semispherical shape of the well traps the bead in the center during measurement. A 20 × 20 matrix scan was made within a 2 mm radius of the center of the well using a CLARIOstar plate reader (BMG Labtech). For the incubation experiments carried out inside the plate reader, LAMPS were incubated in 200 μL of M9 culture medium, taking reads every 20 min using a gain value of 2424. The average value of the 316 reads of the matrix scan was taken as the overall fluorescence of the bead.

End-point single read measurements were used for experiments where LAMPS were incubated externally (cocultures and l-lactate calibration curves). In these experiments, LAMPS were recovered from the medium, rinsed with fresh buffer A (10 mM HEPES, 150 mM NaCl, 20 mM CaCl_2_), and placed in 96-well spheroid microplates containing 200 μL of buffer A. In this case, the reads corresponding to the empty section of the well were not considered for the calculation of the average fluorescence of LAMPS, and the gain was fixed at 1000.

## Supporting Information Available

Supporting methods; measurements of LAB size distribution and polydispersity index calculation; l-lactate titration experiments of the whole-cell biosensor grown in M9-glycerol; measurements of LAMPS coated with chitosan and poly-l-lysine; LAMPS fluorescence signal upon l-lactate spiking and incubation in buffer A; time-course response of LAMPS at different l-lactate concentrations; LAMPS signals after overnight incubation in different culture media; individual contour plots for LAMPS incubated in M9, Ham’s F-12, and CD-CHO at different l-lactate concentrations; LAMPS cocultured with Flp-In CHO and IgG-producing CHO cells; and PCR primers and plasmid sequences (PDF)

## References

[ref1] Gargalo C. L.; Udugama I.; Pontius K.; Lopez P. C.; Nielsen R. F.; Hasanzadeh A.; Mansouri S. S.; Bayer C.; Junicke H.; Gernaey K. V. Towards Smart Biomanufacturing: A Perspective on Recent Developments in Industrial Measurement and Monitoring Technologies for Bio-Based Production Processes. J. Ind. Microbiol. Biotechnol. 2020, 47, 947–964. 10.1007/s10295-020-02308-1. 32895764 PMC7695667

[ref2] Randek J.; Mandenius C. F. On-Line Soft Sensing in Upstream Bioprocessing. Crit. Rev. Biotechnol. 2018, 38, 106–121. 10.1080/07388551.2017.1312271. 28423945

[ref3] Wang X.; Lu X.; Chen J. Development of Biosensor Technologies for Analysis of Environmental Contaminants. Trends Environ. Anal. Chem. 2014, 2, 25–32. 10.1016/j.teac.2014.04.001.

[ref4] Piroozmand F.; Mohammadipanah F.; Faridbod F. Emerging Biosensors in Detection of Natural Products. Synth. Syst. Biotechnol. 2020 5 293–303. 10.1016/j.synbio.2020.08.002. 32954023 PMC7484522

[ref5] Fraser L. A.; Kinghorn A. B.; Dirkzwager R. M.; Liang S.; Cheung Y. W.; Lim B.; Shiu S. C. C.; Tang M. S. L.; Andrew D.; Manitta J.; Richards J. S.; Tanner J. A. A Portable Microfluidic Aptamer-Tethered Enzyme Capture (APTEC) Biosensor for Malaria Diagnosis. Biosens. Bioelectron. 2018, 100, 591–596. 10.1016/j.bios.2017.10.001. 29032164

[ref6] Liu C.; Zeng X.; An Z.; Yang Y.; Eisenbaum M.; Gu X.; Jornet J. M.; Dy G. K.; Reid M. E.; Gan Q.; Wu Y. Sensitive Detection of Exosomal Proteins via a Compact Surface Plasmon Resonance Biosensor for Cancer Diagnosis. ACS Sens. 2018, 3, 1471–1479. 10.1021/acssensors.8b00230. 30019892 PMC8628517

[ref7] Pilas J.; Selmer T.; Keusgen M.; Schöning M. J. Screen-Printed Carbon Electrodes Modified with Graphene Oxide for the Design of a Reagent-Free NAD+-Dependent Biosensor Array. Anal. Chem. 2019, 91, 15293–15299. 10.1021/acs.analchem.9b04481. 31674761

[ref8] Sun A.; Wang W. X. Adenine Deficient Yeast: A Fluorescent Biosensor for the Detection of Labile Zn(II) in Aqueous Solution. Biosens. Bioelectron. 2021, 179, 113075 10.1016/j.bios.2021.113075. 33578113

[ref9] Tang T. C.; Tham E.; Liu X.; Yehl K.; Rovner A. J.; Yuk H.; de la Fuente-Nunez C.; Isaacs F. J.; Zhao X.; Lu T. K. Hydrogel-Based Biocontainment of Bacteria for Continuous Sensing and Computation. Nat. Chem. Biol. 2021, 17, 724–731. 10.1038/s41589-021-00779-6. 33820990 PMC9269716

[ref10] Chaibun T.; Puenpa J.; Ngamdee T.; Boonapatcharoen N.; Athamanolap P.; O’Mullane A. P.; Vongpunsawad S.; Poovorawan Y.; Lee S. Y.; Lertanantawong B. Rapid Electrochemical Detection of Coronavirus SARS-CoV-2. Nat. Commun. 2021, 12, 802 10.1038/s41467-021-21121-7. 33547323 PMC7864991

[ref11] Pan L. H.; Kuo S. H.; Lin T. Y.; Lin C. W.; Fang P. Y.; Yang H. W. An Electrochemical Biosensor to Simultaneously Detect VEGF and PSA for Early Prostate Cancer Diagnosis Based on Graphene Oxide/SsDNA/PLLA Nanoparticles. Biosens. Bioelectron. 2017, 89, 598–605. 10.1016/j.bios.2016.01.077. 26868935

[ref12] Sempionatto J. R.; Lin M.; Yin L.; De E.; Pei K.; Sonsa-ard T.; Silva A. N. D. L.; Khorshed A. A.; Zhang F.; Tostado N.; Xu S.; Wang J. An Epidermal Patch for the Simultaneous Monitoring of Haemodynamic and Metabolic Biomarkers. Nat. Biomed. Eng. 2021, 5, 737 10.1038/s41551-021-00685-1. 33589782

[ref13] Collins J. J.; Gardner T. S.; Cantor C. R. Construction of a Genetic Toggle Switch in *Escherichia coli*. Nature 2000, 403, 339–342. 10.1038/35002131. 10659857

[ref14] Dey A.; Barik D. Emergent Bistable Switches from the Incoherent Feed-Forward Signaling of a Positive Feedback Loop. ACS Synth. Biol. 2021, 10, 3117–3128. 10.1021/acssynbio.1c00373. 34694110

[ref15] Wang B.; Kitney R. I.; Joly N.; Buck M. Engineering Modular and Orthogonal Genetic Logic Gates for Robust Digital-like Synthetic Biology. Nat. Commun. 2011, 2, 508 10.1038/ncomms1516. 22009040 PMC3207208

[ref16] Wang B.; Barahona M.; Buck M. Engineering Modular and Tunable Genetic Amplifiers for Scaling Transcriptional Signals in Cascaded Gene Networks. Nucleic Acids Res. 2014, 42, 9484–9492. 10.1093/nar/gku593. 25030903 PMC4132719

[ref17] Prindle A.; Samayoa P.; Razinkov I.; Danino T.; Tsimring L. S.; Hasty J. A Sensing Array of Radically Coupled Genetic “Biopixels. Nature 2012, 481, 39–44. 10.1038/nature10722. PMC3259005 22178928

[ref18] Wan X.; Volpetti F.; Petrova E.; French C.; Maerkl S. J.; Wang B. Cascaded Amplifying Circuits Enable Ultrasensitive Cellular Sensors for Toxic Metals. Nat. Chem. Biol. 2019, 15, 540–548. 10.1038/s41589-019-0244-3. 30911179

[ref19] Zhang F.; Carothers J. M.; Keasling J. D. Design of a Dynamic Sensor-Regulator System for Production of Chemicals and Fuels Derived from Fatty Acids. Nat. Biotechnol. 2012, 30, 354–359. 10.1038/nbt.2149. 22446695

[ref20] Lin M.; Song P.; Zhou G.; Zuo X.; Aldalbahi A.; Lou X.; Shi J.; Fan C. Electrochemical Detection of Nucleic Acids, Proteins, Small Molecules and Cells Using a DNA-Nanostructure-Based Universal Biosensing Platform. Nat. Protoc. 2016, 11, 1244–1263. 10.1038/nprot.2016.071. 27310264

[ref21] Komatsu N.; Aoki K.; Yamada M.; Yukinaga H.; Fujita Y.; Kamioka Y.; Matsuda M. Development of an Optimized Backbone of FRET Biosensors for Kinases and GTPases. Mol. Biol. Cell 2011, 22, 4647–4656. 10.1091/mbc.E11-01-0072. 21976697 PMC3226481

[ref22] Li D.; Song S.; Fan C. Target-Responsive Structural Switching for Nucleic Acid-Based Sensors. Acc. Chem. Res. 2010, 43, 631–641. 10.1021/ar900245u. 20222738

[ref23] Goers L.; Ainsworth C.; Goey C. H.; Kontoravdi C.; Freemont P. S.; Polizzi K. M. Whole-Cell *Escherichia coli* Lactate Biosensor for Monitoring Mammalian Cell Cultures during Biopharmaceutical Production. Biotechnol. Bioeng. 2017, 114, 1290–1300. 10.1002/bit.26254. 28112405 PMC5412874

[ref24] Trantidou T.; Dekker L.; Polizzi K.; Ces O.; Elani Y. Functionalizing Cell-Mimetic Giant Vesicles with Encapsulated Bacterial Biosensors. Interface Focus 2018, 8, 20180024 10.1098/rsfs.2018.0024. 30443325 PMC6227772

[ref25] Anderson J. C. Anderson’s Promoter Collection. http://parts.igem.org/Promoters/Catalog/Anderson.

[ref26] Ladet S.; David L.; Domard A. Multi-Membrane Hydrogels. Nature 2008, 452, 76–79. 10.1038/nature06619. 18322531

[ref27] Mao A. S.; Özkale B.; Shah N. J.; Vining K. H.; Descombes T.; Zhang L.; et al. Programmable Microencapsulation for Enhanced Mesenchymal Stem Cell Persistence and Immunomodulation. Proc. Natl. Acad. Sci. U.S.A. 2019, 116, 15392–15397. 10.1073/pnas.1819415116. 31311862 PMC6681761

[ref28] Simó G.; Fernández-Fernández E.; Vila-Crespo J.; Ruipérez V.; Rodríguez-Nogales J. M. Research Progress in Coating Techniques of Alginate Gel Polymer for Cell Encapsulation. Carbohydr. Polym. 2017, 170, 1–14. 10.1016/j.carbpol.2017.04.013. 28521974

[ref29] Li P.; Müller M.; Chang M. W.; Frettlöh M.; Schönherr H. Encapsulation of Autoinducer Sensing Reporter Bacteria in Reinforced Alginate-Based Microbeads. ACS Appl. Mater. Interfaces 2017, 9, 22321–22331. 10.1021/acsami.7b07166. 28627870 PMC5741077

[ref30] Kim B. J.; Park T.; Moon H. C.; Park S. Y.; Hong D.; Ko E. H.; Kim J. Y.; Hong J. W.; Han S. W.; Kim Y. G.; Choi I. S. Cytoprotective Alginate/Polydopamine Core/Shell Microcapsules in Microbial Encapsulation. Angew. Chem., Int. Ed. 2014, 53, 14443–14446. 10.1002/anie.201408454. 25354197

[ref31] Ball V. Polydopamine Nanomaterials: Recent Advances in Synthesis Methods and Applications. Front. Bioeng. Biotechnol. 2018, 6, 109 10.3389/fbioe.2018.00109. 30175095 PMC6108306

[ref32] Mørch Y. A.; Donati I.; Strand B. L.; Skjåk-Bræk G. Effect of Ca2+, Ba2+, and Sr2+ on Alginate Microbeads. Biomacromolecules 2006, 7, 1471–1480. 10.1021/bm060010d. 16677028

[ref33] Goers L.; Freemont P.; Polizzi K. M. Co-Culture Systems and Technologies: Taking Synthetic Biology to the next Level. J. R. Soc. Interface 2014, 11, 20140065 10.1098/rsif.2014.0065. 24829281 PMC4032528

[ref34] Burmeister A.; Grünberger A. Microfluidic Cultivation and Analysis Tools for Interaction Studies of Microbial Co-Cultures. Curr. Opin. Biotechnol. 2020, 62, 106–115. 10.1016/j.copbio.2019.09.001. 31715386

[ref35] Zhu H.; Meng H.; Zhang W.; Gao H.; Zhou J.; Zhang Y.; Li Y. Development of a Longevous Two-Species Biophotovoltaics with Constrained Electron Flow. Nat. Commun. 2019, 10, 4282 10.1038/s41467-019-12190-w. 31537786 PMC6753107

[ref36] Johnston T. G.; Yuan S. F.; Wagner J. M.; Yi X.; Saha A.; Smith P.; Nelson A.; Alper H. S. Compartmentalized Microbes and Co-Cultures in Hydrogels for on-Demand Bioproduction and Preservation. Nat. Commun. 2020, 11, 563 10.1038/s41467-020-14371-4. 32019917 PMC7000784

[ref37] Shahab R. L.; Brethauer S.; Davey M. P.; Smith A. G.; Vignolini S.; Luterbacher J. S.; Studer M. H. A Heterogeneous Microbial Consortium Producing Short-Chain Fatty Acids from Lignocellulose. Science 2020, 369, eabb1214 10.1126/science.abb1214. 32855308

[ref38] Smith M. J.; Francis M. B. Improving Metabolite Production in Microbial Co-Cultures Using a Spatially Constrained Hydrogel. Biotechnol. Bioeng. 2017, 114, 1195–1200. 10.1002/bit.26235. 27943258

[ref39] Xu Z.; Wang S.; Zhao C.; Li S.; Liu X.; Wang L.; Li M.; Huang X.; Mann S. Photosynthetic Hydrogen Production by Droplet-Based Microbial Micro-Reactors under Aerobic Conditions. Nat. Commun. 2020, 11, 5985 10.1038/s41467-020-19823-5. 33239636 PMC7689460

[ref40] Kotula J. W.; Kerns S. J.; Shaket L. A.; Siraj L.; Collins J. J.; Way J. C.; Silver P. A. Programmable Bacteria Detect and Record an Environmental Signal in the Mammalian Gut. Proc. Natl. Acad. Sci. U.S.A. 2014, 111, 4838–4843. 10.1073/pnas.1321321111. 24639514 PMC3977281

[ref41] Werlang C.; Cárcarmo-Oyarce G.; Ribbeck K. Engineering Mucus to Study and Influence the Microbiome. Nat. Rev. Mater. 2019, 4, 134–145. 10.1038/s41578-018-0079-7. PMC11906034 40084234

[ref42] Erickson A. K.; Jesudhasan P. R.; Mayer M. J.; Narbad A.; Winter S. E.; Pfeiffer J. K. Bacteria Facilitate Enteric Virus Co-Infection of Mammalian Cells and Promote Genetic Recombination. Cell Host Microbe 2018, 23, 77.e5–88.e5. 10.1016/j.chom.2017.11.007. 29290575 PMC5764776

[ref43] Iwama T.; Fujiya M.; Konishi H.; Tanaka H.; Murakami Y.; Kunogi T.; Sasaki T.; Takahashi K.; Ando K.; Ueno N.; Kashima S.; Moriichi K.; Tanabe H.; Okumura T. Bacteria-derived Ferrichrome Inhibits Tumor Progression in Sporadic Colorectal Neoplasms and Colitis-associated Cancer. Cancer Cell Int. 2021, 21, 21 10.1186/s12935-020-01723-9. 33407519 PMC7789586

[ref44] Chiang C. J.; Huang P. H. Metabolic Engineering of Probiotic *Escherichia coli* for Cytolytic Therapy of Tumors. Sci. Rep. 2021, 11, 5853 10.1038/s41598-021-85372-6. 33712706 PMC7971005

[ref45] Fedorec A.; Karkaria B.; Sulu M.; Barnes C. Single Strain Control of Microbial Consortia. Nat. Commun. 2019, 4, 1977 10.1038/s41467-021-22240-x. PMC8010080 33785746

[ref46] Thor D.; Xiao N.; Yu R.; Jivan A.; Cha B. Induction of EGFP Expression in Pichia Pastoris during Co-Culture with Human Endothelial Cell Line. J. Microbiol. Methods 2019, 161, 28–34. 10.1016/j.mimet.2019.04.006. 30995456

[ref47] Le H. H. M.; Vang D.; Amer N.; Vue T.; Yee C.; Kaou H.; Harrison J. S.; Xiao N.; Lin-Cereghino J.; Lin-Cereghino G. P.; Thor D. Enhancement of Cell Proliferation and Motility of Mammalian Cells Grown in Co-Culture with *Pichia pastoris* Expressing Recombinant Human FGF-2. Protein Expr. Purif. 2020, 176, 105724 10.1016/j.pep.2020.105724. 32846209

[ref48] Kunjapur A. M.; Napolitano M. G.; Hysolli E.; Noguera K.; Appleton E. M.; Schubert M. G.; Jones M. A.; Iyer S.; Mandell D. J.; Church G. M. Synthetic Auxotrophy Remains Stable after Continuous Evolution and in Co-Culture with Mammalian Cells. bioRxiv 2020, 18, 2020.09.27.315804 10.1101/2020.09.27.315804. PMC11060021 34215581

[ref49] Velasco D.; Tumarkin E.; Kumacheva E. Microfluidic Encapsulation of Cells in Polymer Microgels. Small 2012, 8, 1633–1642. 10.1002/smll.201102464. 22467645

[ref50] Paredes Juárez G. A.; Spasojevic M.; Faas M. M.; de Vos P. Immunological and Technical Considerations in Application of Alginate-Based Microencapsulation Systems. Front. Bioeng. Biotechnol. 2014, 2, 26 10.3389/fbioe.2014.00026. 25147785 PMC4123607

[ref51] Mawad A.; Helmy Y. A.; Shalkami A.-G.; Kathayat D.; Rajashekara G. *E. coli* Nissle Microencapsulation in Alginate-Chitosan Nanoparticles and Its Effect on *Campylobacter jejuni* in Vitro. Appl. Microbiol. Biotechnol. 2018, 102, 10675–10690. 10.1007/s00253-018-9417-3. 30302522

[ref52] Belkin S.; Wang B. Sense and Sensibility: Of Synthetic Biology and the Redesign of Bioreporter Circuits. Microb. Biotechnol. 2021, 103–106. 10.1111/1751-7915.13955. 34689402 PMC8719829

[ref53] Hossain G. S.; Saini M.; Miyake R.; Ling H.; Chang M. W. Genetic Biosensor Design for Natural Product Biosynthesis in Microorganisms. Trends Biotechnol. 2020 38 797–810. 10.1016/j.tibtech.2020.03.013. 32359951

[ref54] Dixon T. A.; Williams T. C.; Pretorius I. S. Sensing the Future of Bio-Informational Engineering. Nat. Commun. 2021, 12, 388 10.1038/s41467-020-20764-2. 33452260 PMC7810845

