# Supplementary material for: Polymer Encapsulation of Bacterial Biosensors Enables Coculture with Mammalian Cells
Source: ACS Synth Biol. 2022 Mar 4;11(3):1303–12. doi: 10.1021/acssynbio.1c00577 (PMC9007569; doi:10.1021/acssynbio.1c00577)
Supplement: Supplementary file 1 — sb1c00577_si_001.pdf [file sb1c00577_si_001.pdf]

Polymer encapsulation of bacterial biosensors enables co-culture with mammalian cells

Ignacio Moya-Ramírez [1,2,3], Pavlos Kotidis[1], Masue Marbiah[1,2], Juhyun Kim[1,2], Cleo Kontoravdi[1], Karen Polizzi[1,2,*]

[1] Department of Chemical Engineering, Imperial College London, London, SW7 2AZ, United Kingdom

[2] Imperial College Centre for Synthetic Biology, Imperial College London, London, SW7 2AZ, United Kingdom

[3] Present address: Department of Chemical Engineering, Universidad de Granada, Departamento de Ingeniería Química, Spain, 18071 Avda. Fuentenueva s/n, Granada.

* To whom correspondence should be addressed: k.polizzi@imperial.ac.uk

1. Supplementary methods

1.1. Construction and characterization of the L-lactate-biosensing plasmid.

The starting L-lactate-biosensing plasmid used in this work (pLac) is described in Trantidou et al.,[1] and is a modification of the original plasmid design of Goers et al.[2], where the native lldPRD promoter from *E. coli* was exchanged for a synthetic promoter consisting of the constitutive J23117 promoter flanked by the O1 and O2 sites of the natural promoter (Figure 1a). For the present work, the promoters J23100 and J23118 were used individually in place of the Hyperspank promoter K143015 to control the expression of the lldR transcription factor and the selection marker was changed from β-lactamase (ampicillin resistance) to kanamycin phosphotransferase. The aim of these changes was to achieve a better dose response of the system and avoid the need to add IPTG to the current experimental set up to induce lldR expression.

For the Hyperspank promoter substitution, the pLac plasmid was amplified by inverse PCR with primers P1 and P2 upstream and downstream of the promoter. The PCR product was gel extracted, purified and digested with BamHI HF and XhoI restriction enzymes (New England Biolabs, UK) and subsequently purified. The J23100 and J23118 promoter inserts were prepared by annealing primers P3 and P4 and P5 and P6, respectively. The primers were dissolved in annealing buffer (10 mM Tris-HCl, 50 mM NaCl, 1 mM EDTA, pH 7.5), mixed in equal volumes, warmed to 99ºC in a heat block and left to cool down to room temperature. The resulting dsDNA inserts contained unpaired ends complementary to the BamHI and XhoI restriction sites and were ligated to the previously obtained backbone using T4 ligase (Promega, UK) to obtain the plasmids pLact-100 and pLact-118.

E. coli DH5α cells were used for expression and characterization of all the plasmids constructs. A colony of freshly transformed cells carrying the plasmid of interest was inoculated into 5 mL of LB medium supplemented with 37.5 mg/L of kanamycin and grown at 37°C with shaking at 250 rpm. After 8-10 h, 100 µL of this starter culture was used to inoculate 10 mL of fresh M9 medium with 37.5 mg/L of kanamycin and grown for 16 h under the same conditions. Fresh M9 medium was inoculated from this culture to a starting OD of 0.05 in 200 µL cultures in 96 well flat bottom plates (Corning, USA). The plate was kept at room temperature for 2h 30 min to simulate the preparation time of the LAMPS beads. L-lactate and IPTG were added to the desired concentrations after this incubation step. The plates were covered with a Breath Easy membrane (Sigma) and incubated in a CLARIOstar plate reader (BMG Labtech, UK) at 37°C and 500 rpm, taking fluorescence reads every 15 min with λ_{ex}=470 nm and λ_{em}=515 nm for the detection of GFP. The fluorescence measurements were normalized by the corresponding OD_{600} measurement.

1.2. Coating with chitosan (CH) and poly-dopamine (PD)

The coating with PD followed the same protocol described in the main text, using a 5 mg/mL dopamine hydrochloride solution. The incubation was extended either to 3.5 h or 12 h.

Coating with CH followed the method by Gåserød et al.[3] A 1.5 g/L chitosan solution was prepared in a solution of 0.02M CH_3COONa and 0.1 M $CaCl_2$. The mix was acidified with HCl to pH 4, and upon dissolution of the chitosan, the pH was adjusted to 6 with NaOH and filter sterilized. The chistosan solution was used in a 1 hour incubation step for the first layer and 2 hours for the second. The rest of the steps were as described in the main text.

1.3. Analytical methodology

- **Culture viability and cell density**

Culture viability and cell density were measured in a NucleoCounter® NC-250 (Chemometec, Germany), by staining 95 µL of cell culture with 5 µL of solution C-18 (acridine orange+DAPI, Chemometec). Prior to measurements adherent CHO cells were washed with 2 mL of PBS, incubated with 0.5 mL of prewarmed 0.05% trypsin solution at 37 °C for 5 min on a rocking platform, followed by the addition of 1 mL of neutralizing solution (10% FBS in PBS). Suspension cells were used directly for measurements.

- **L-lactate concentration**

L(+)-lactate concentration in the culture media was measured with an enzyme-based assay kit (MAK064, Sigma, UK) following the instruction provided by the manufacturer. For sample preparation, cells and insoluble material were removed from a 1 mL culture sample by centrifugation at 13000 g for 10 min and filtering with a 5 kDa MWCO spin filter (Vivaspin 500, Sartorius) to remove any lactate dehydrogenase contamination. Prepared samples were kept at -20 °C until analysis. L-lactate concentration was measured spectrophotometrically using absorbance at 570 nm. Samples were diluted as needed to have a concentration between 0.05

and 0.1 µmol/mL for the enzymatic reaction. A calibration curve with a standard of L-lactate was prepared to enable quantification.

- **Bacterial escape from LAMPS**

To test possible escape of *E. coli* cells from the LAMPS during incubation, 100 µL of culture medium was plated on LB-agar plates containing 37.5 mg/L kanamycin and incubated overnight at 37ºC to assess colony forming units (CFU). Plates were analysed in at least duplicate for each timepoint during the co-culture experiment and the optimization of the encapsulation protocol.

2. Supplementary results and discussion

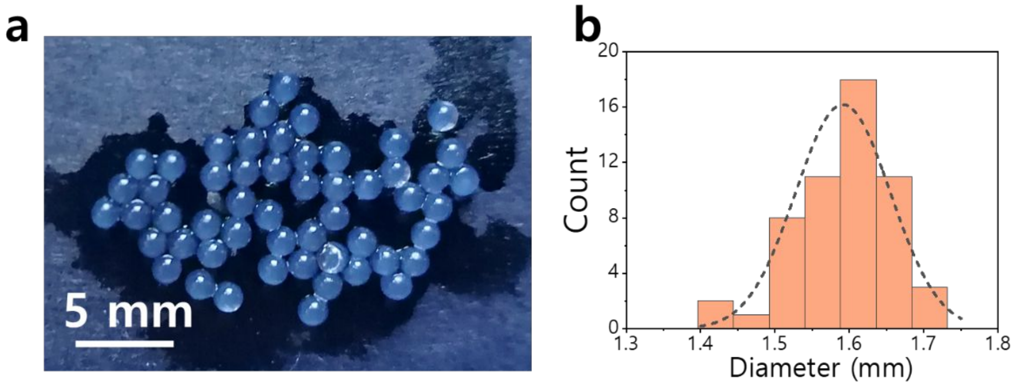

Figure S1. **a)** LAB alginate cores. b) Histogram showing the distribution of the diameter of the LAB cores with a Gaussian fit. The polydispersity index calculated as the square of the quotient of the standard deviation over the mean diameter of the sample of n=54 was 0.00157.

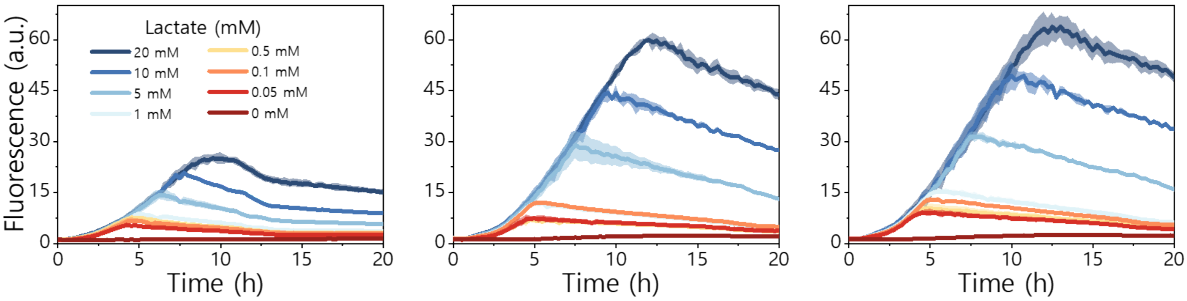

Figure S2. L-lactate titration experiments with liquid cultures of *E. coli* carrying one of the three different L-lactate-sensing plasmids: pLac, pLac_100 or pLac_118. Cells were grown in M9 with glycerol 0.4 % as carbon source. **The lines represent the mean and shading indicates the standard deviation (n=2).** The equivalent experiment with glucose is included in the main text (Figure 1e).

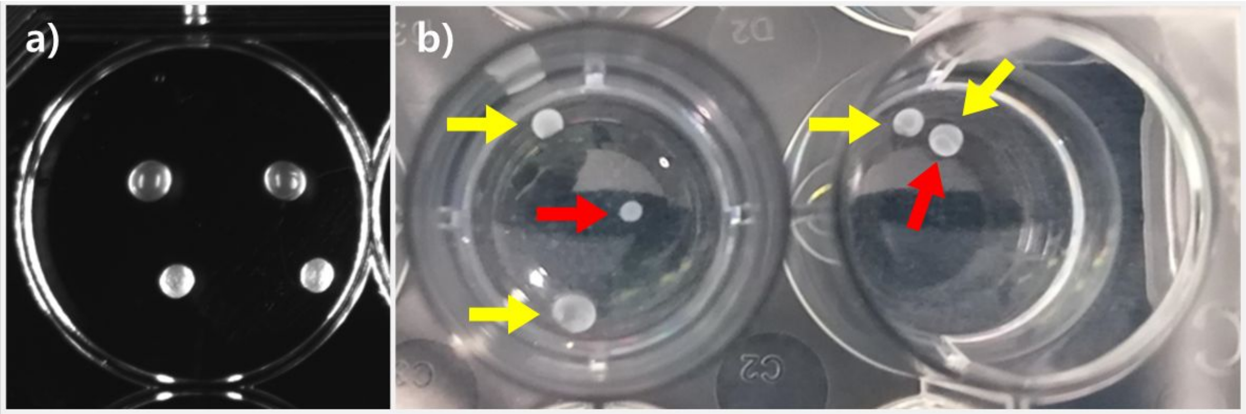

Figure S3. Chitosan and poly-L-lysine coated LAMPS after incubation. **a)** Chitosan (CH, top) and poly-L-lysine (PLL, bottom) coated LAMPS after ON incubation in buffer A. The effect of the coating can be observed on the transparency and the size of the beads. CH-coated beads were more transparent and, upon disruption and plating on agar, showed higher CFU. On the other hand, PLL-coated beads resisted the disruption in buffer B (0.1 M EDTA and 0.2 M potassium citrate) for 1 h and required mechanical disruption to release the encapsulated bacteria. In addition, PLL seemed to prevent swelling of the bead during the incubation. This observation is also compatible with a shrinking described for PLL-coated beads, that could be due to the neutralization of negative charges of the alginate polymer.[4] **b)** LAMPS with two layers of chitosan after their ON incubation in M9. The detachment of the coating layers (yellow arrows) and shells (red arrows) leaving the inner shell exposed can be observed.

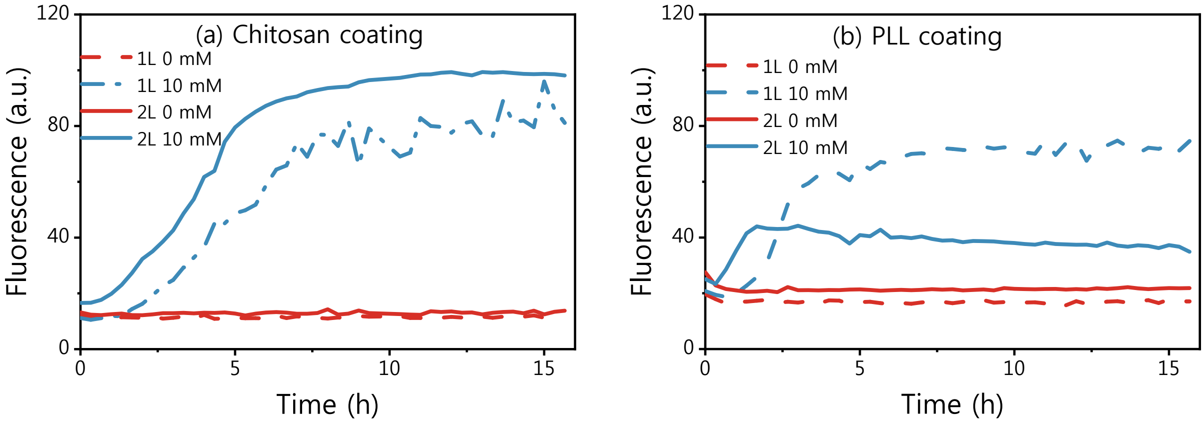

Figure S4 LAMPS fluorescence signal upon L-lactate spiking and incubation in buffer A, for LAMPS with one and two layers (dashed and straight lines respectively), prepared with CH (a) and PLL (b). Here, the fresh M9 used for the resuspension of the cells during the preparation of the LAMPS is the only nutrient source available, therefore a higher number of layers will reduce the diffusion rate of the nutrients outside the bead.

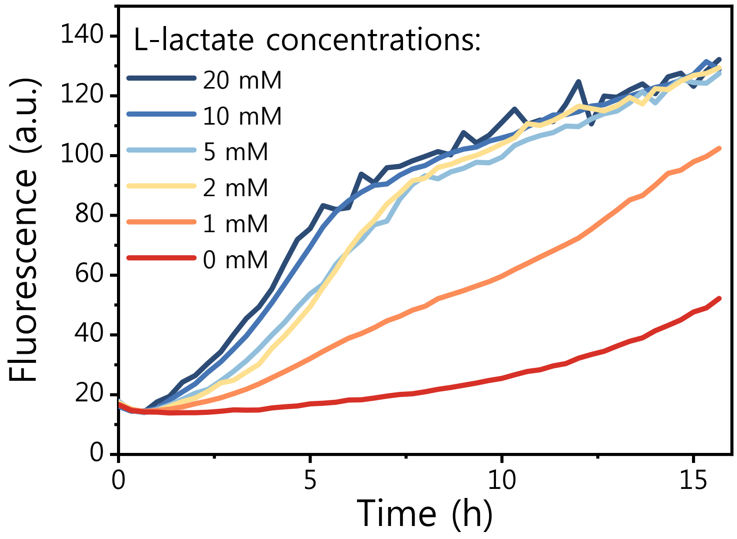

Figure S5. Response of LAMPS beads over time when incubated in a 96-well plate at different L-lactate concentrations. The signal was proportional to the concentration of L-lactate during the first 5 hours approximately. For longer periods, it saturates at L-lactate concentrations above 2 mM. Datapoints at 1, 2.6 and 5 hours were extracted from these data and are presented in Figure 3.a.

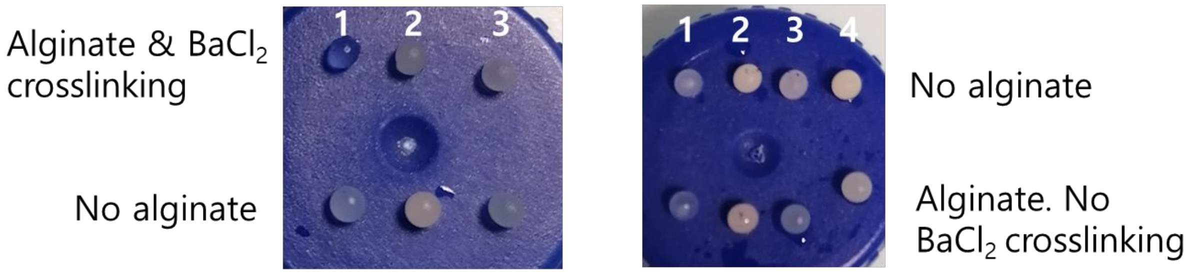

Figure S6. LAMPS after overnight incubation in different culture media: columns 1, 2 3 and 4 correspond to M9, CD-CHO, Ham's F12 and Opti-MEM respectively. PLL coated LAMPS are compared to beads covered with an additional alginate layer, either crosslinked with $BaCl_2$ (left) or not (right).

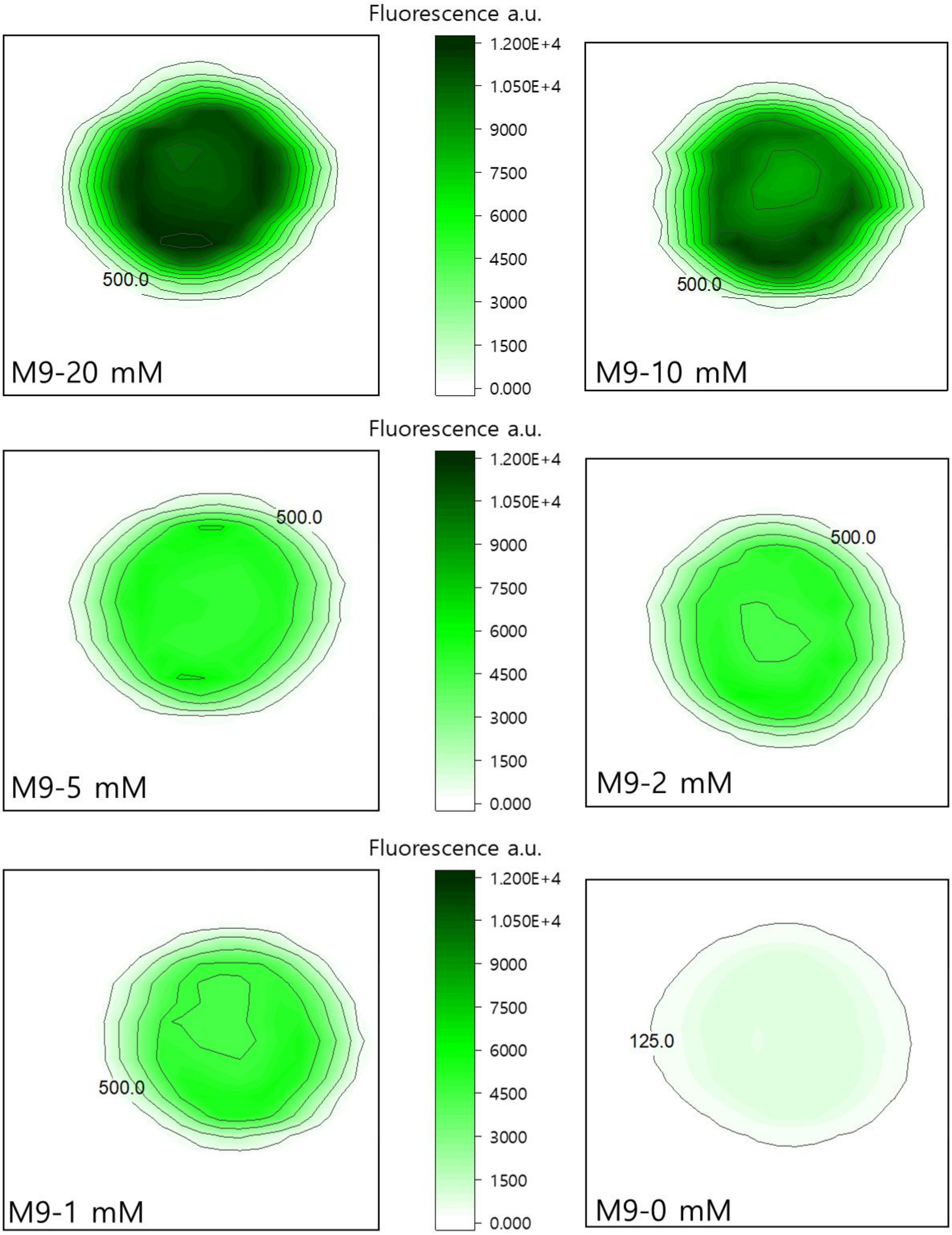

Figure S7. Contour plots for LAMPS incubated in M9 for 20 h at different L-lactate concentrations, used to build the transfer function shown in Figure 4.a. The fluorescence threshold delimiting the edge of the LAMPS and the empty section of the well (see supplementary methods 1.3) is indicated for each measurement.

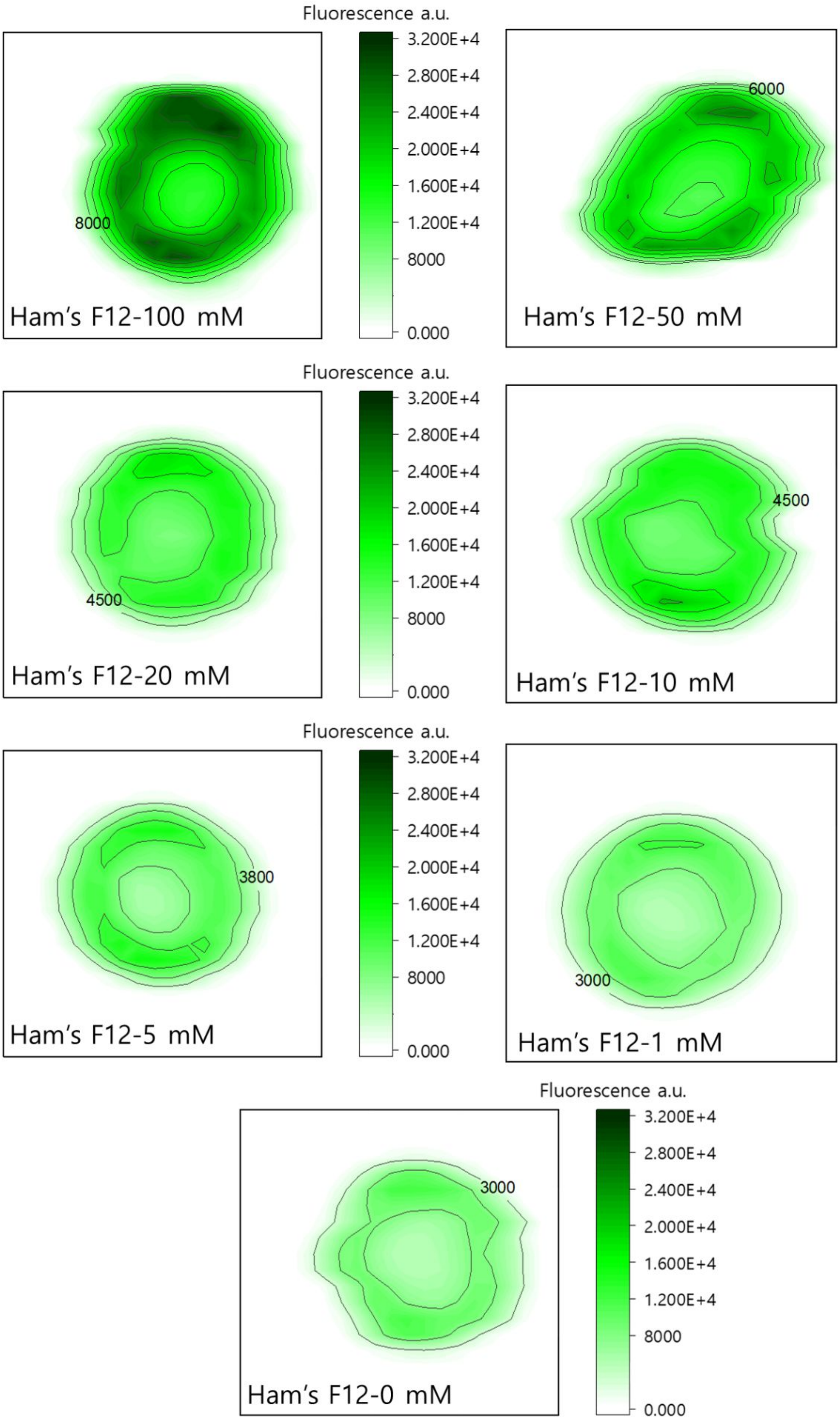

Figure S8. Contour plots for LAMPS incubated in Ham's F-12 for 20 h at different L-lactate concentrations, used to build the transfer function shown in Figure 4.a. The fluorescence threshold is indicated for each bead.

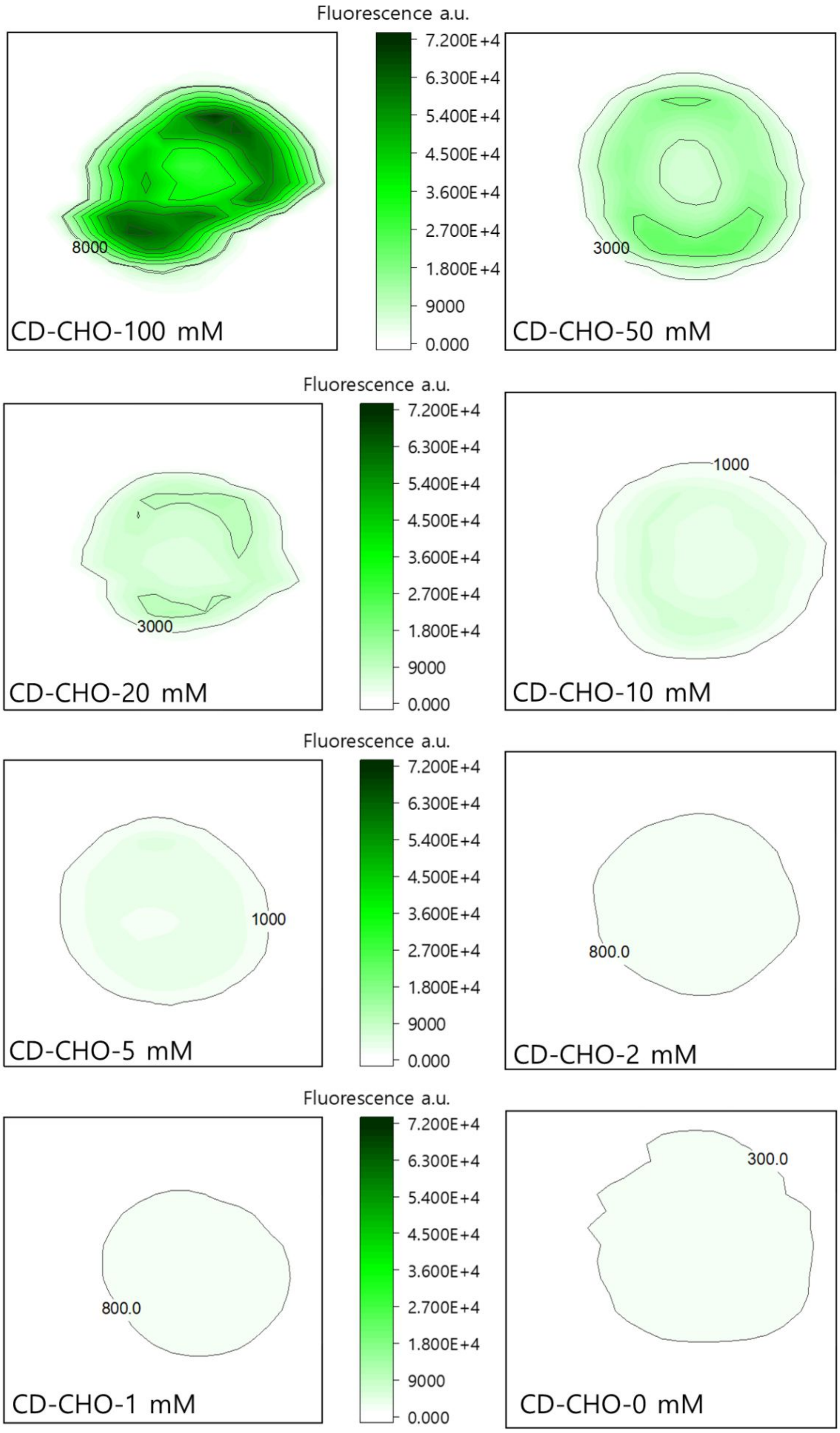

Figure S9. Contour plots for LAMPS incubated in CD-CHO for 20 h at different L-lactate concentrations, used to build the transfer function shown in Figure 4.a. The fluorescence threshold is indicated for each bead.

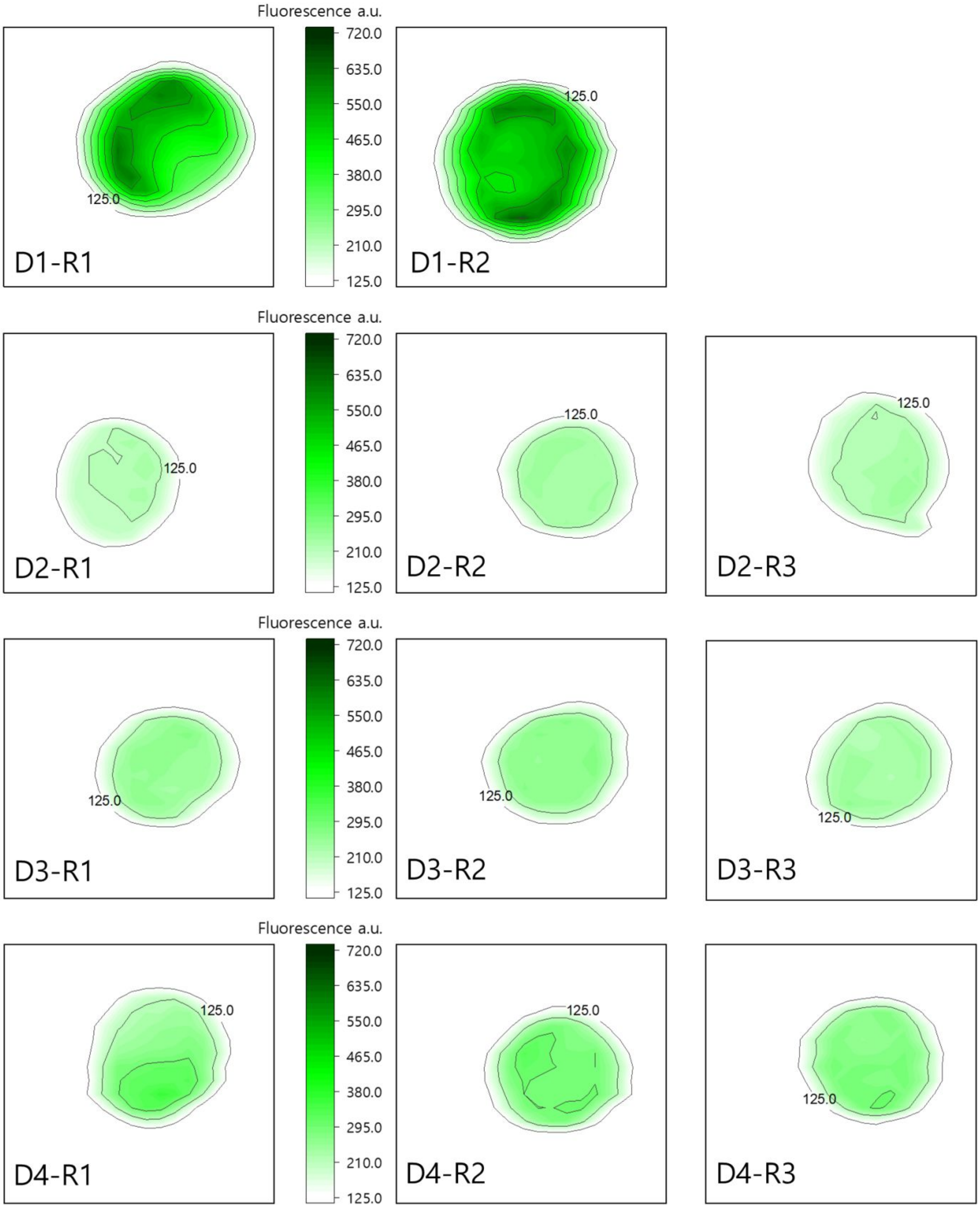

Figure S10. Contour plots for LAMPS co-cultured for 20 h with Flp-In CHO cells that previously were grown as a monoculture for 1 to 4 days. Duplicate or triplicate LAMPS beads were analysed for each day of the experiment. The fluorescence threshold is indicated for each bead.

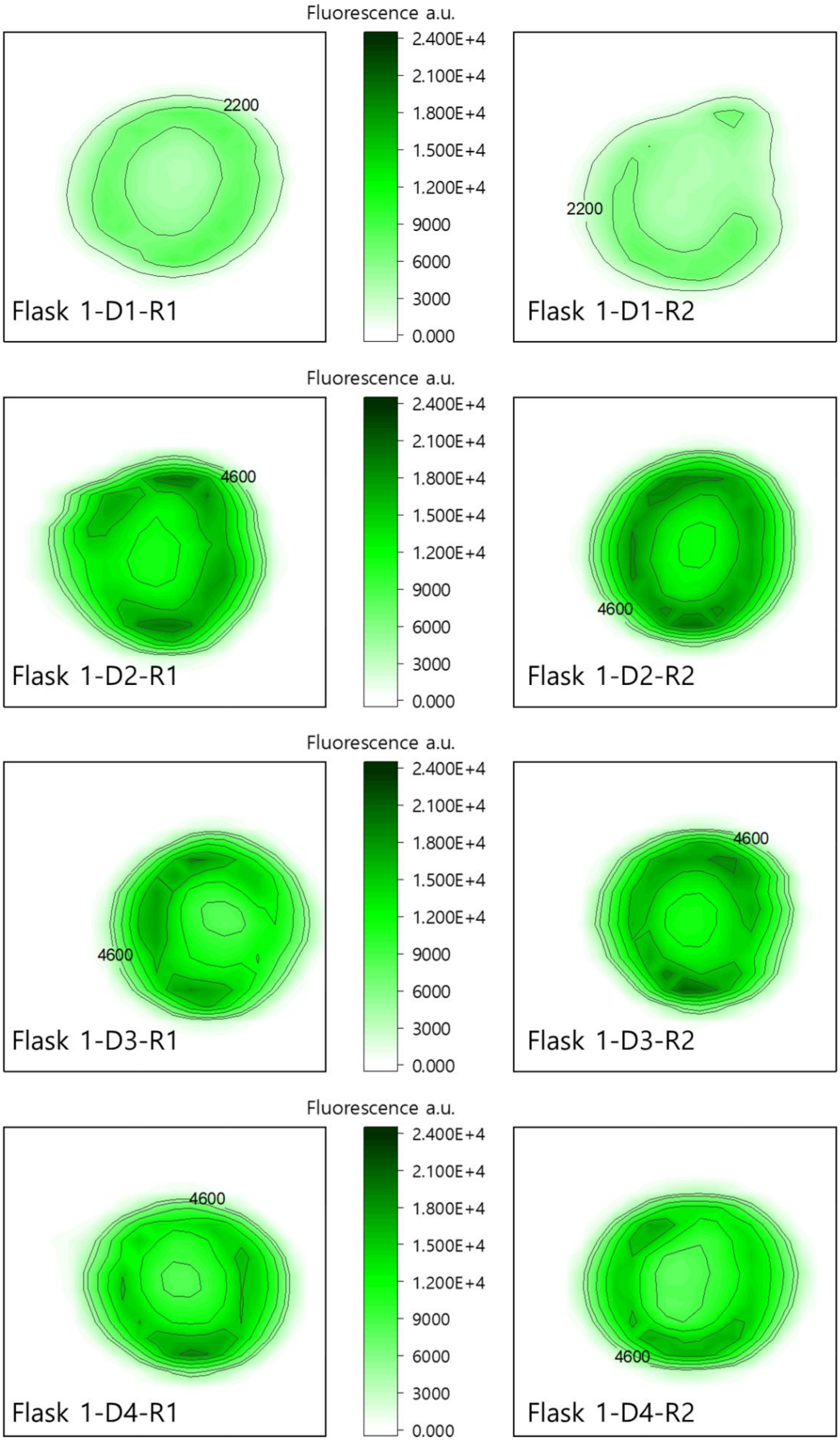

Figure S11. Contour plots for LAMPS co-cultured for 20 h with IgG-producing CHO cells that previously were grown as a monoculture for 1 to 4 days. For each day, two LAMPS beads were

co-cultured in each flask. The fluorescence threshold is indicated for each bead. Results for flask one.

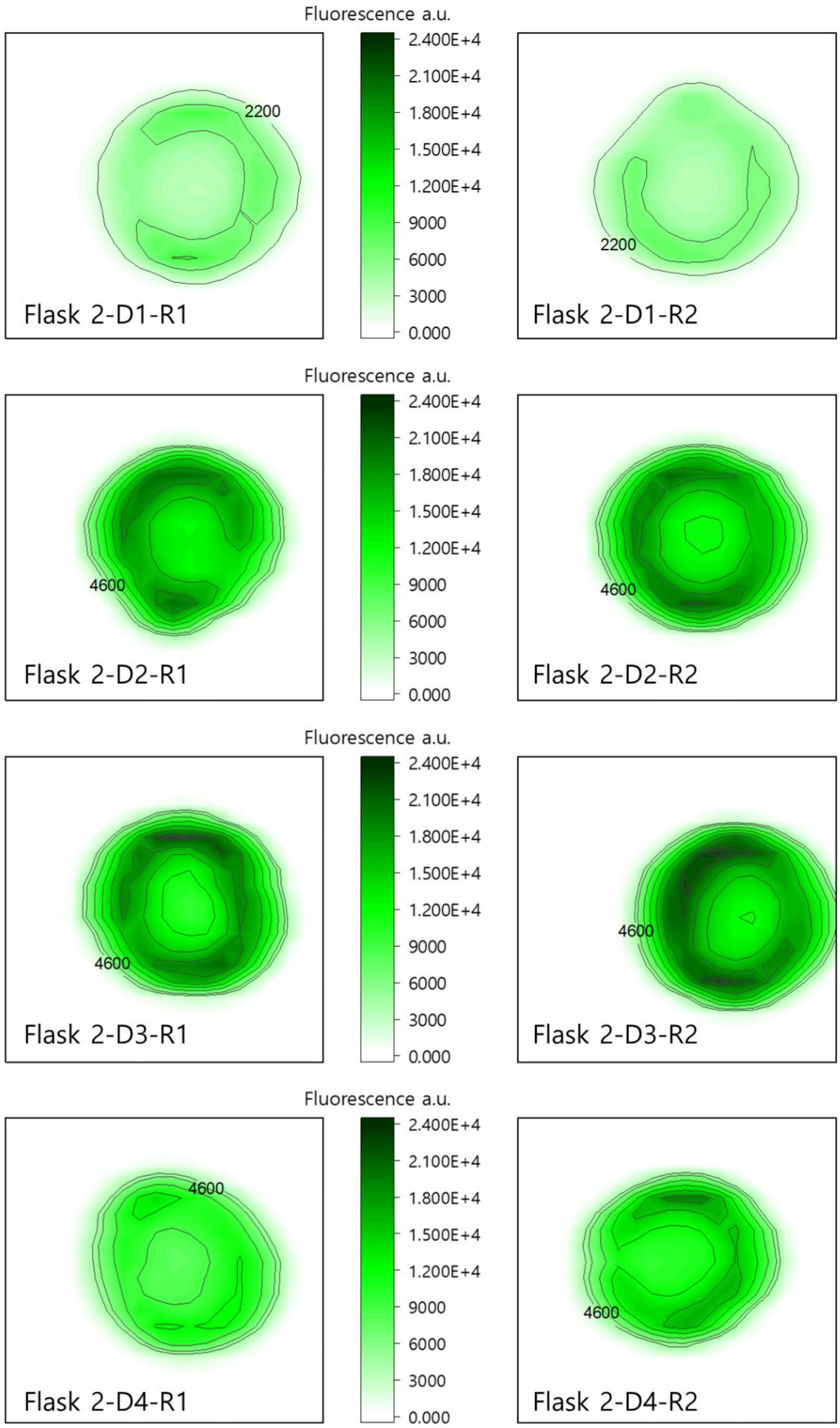

Figure S. 10 (continuation). Results for flask 2.

3. Primers and plasmid sequences

Name	Orientation	Sequence
P1	Forward	tgcaccggatccttactagagaaaggtggtgaatac
P2	Reverse	acatttaccctcgagctctagt
P3	Forward	tcgagttgacggctagctcagtcctaggtacagtgctagcg
P4	Reverse	gatccgctagcactgtacctaggactgagctagccgtcaac
P5	Forward	tcgagttgacggctagctcagtcctaggtattgtgctagcg
P6	Reverse	gatccgctagcacaatacctaggactgagctagccgtcaac

pLac:

gaattcgcggccgcttctagagctttaccagacatctccccccacaagaattggccctaccaattcttcgcttatctgacctctggttcacaattt
cccaattaaaactcacatcaatgttgacagctagctcagtcctagggattgtgctagctcattatccctacacaacacaattggcagtgccactt
ttacacaacgtgtgacaaggagatgagcaacagactcattacacgatgtgcgtggactcctactagagaaagaggagaaatactagatgc
gtaaaggagaagaacttttcactggagttgtcccaattcttgttgaattagatggtgatgttaatgggcacaaattttctgtcagtggagagg
gtgaaggtgatgcaacatacggaaaacttacccttaaatttatttgcactactggaaaactacctgttccatggccaacacttgtcactactttc
ggttatggtgttcaatgctttgcgagatacccagatcatatgaaacagcatgactttttcaagagtgccatgcccgaaggttatgtacaggaa
agaactatatttttcaaagatgacgggaactacaagacacgtgctgaagtcaagtttgaaggtgatacccttgttaatagaatcgagttaaaa
ggtattgattttaaagaagatggaaacattcttggacacaaattggaatacaactataactcacacaatgtatacatcatggcagacaaacaa
aagaatggaatcaaagttaacttcaaaattagacacaacattgaagatggaagcgttcaactagcagaccattatcaacaaaatactccaatt
ggcgatggccctgtccttttaccagacaaccattacctgtccacacaatctgccctttcgaaagatcccaacgaaaagagagaccacatggtc
cttcttgagtttgtaacagctgctgggattacacatggcatggatgaactatacaaataataatactagagccaggcatcaaataaaacgaaa
ggctcagtcgaaagactgggcctttcgttttatctgttgtttgtcggtgaacgctctctactagagtcacactggctcaccttcgggtgggcctt
tctgcgtttatatactagagctcgagggtaaatgtgagcactcacaattcattttgcaaaagttgttgactttatctacaaggtgtggcataatg
tgtgtaattgtgagcggataacaatttactagagaaaggtggtgaatactagagatgattgttttacccagacgcctgtcagacgaggttgc
cgatcgtgtgcgggcgctgattgatgaaaaaaacctggaagcgggcatgaagttgcccgctgagcgccaactggcgatgcaactcggc
gtatcacgtaattcactgcgcgaggcgctggcaaaactggtgagtgaaggcgtgctgctcagtcgacgcggcggcgggacgtttattcgc
tggcgtcatgacacatggtcggagcaaaacatcgtccagccgctaaaaacactgatggccgatgatccggattacagtttcgatattctgga
agcccgctacgccattgaagccagcaccgcatggcatgcggcaatgcgcgccacacctggcgacaaagaaaagattcagctttgctttga
agcaacgctaagtgaagacccggatatcgcctcacaagcggacgttcgttttcatctggcgattgccgaagcctcacataacatcgtgctgc
tgcaaaccatgcgcggtttcttcgatgtcctgcaatcctcagtgaagcatagccgtcagcggatgtatctggtgccaccggtttttttcacaact
gaccgaacaacatcaggctgtcattgacgccatttttgccggtgatgctgacggggcgcgtaaagcaatgatggcgcaccttagttttgttc
acaccaccatgaaacgattcgatgaagatcaggctcgccacgcacggattacccgcctgcccggtgagcataatgagcattcgagggaga
aaaacgcatgatactagagtcacactggctcaccttcgggtgggcctttctgcgtttatatactagagagagaatataaaaagccagattatt
aatccggctttttttattattttactagagtactagtagcggccgctgcaggcttcctcgctcactgactcgctgcgctcggtcgttcggctgcgg
cgagcggtatcagctcactcaaaggcggtaatacggttatccacagaatcaggggataacgcaggaaagaacatgtgagcaaaaggcc
agcaaaaggccaggaaccgtaaaaaggccgcgttgctggcgtttttccataggctccgcccccctgacgagcatcacaaaaatcgacgctc
aagtcagaggtggcgaaacccgacaggactataaagataccaggcgtttccccctggaagctccctcgtgcgctctcctgttccgaccctgc
cgcttaccggatacctgtccgcctttctcccttcgggaagcgtggcgctttctcatagctcacgctgtaggtatctcagttcggtgtaggtcgtt
cgctccaagctgggctgtgtgcacgaaccccccgttcagcccgaccgctgcgccttatccggtaactatcgtcttgagtccaacccggtaag
acacgacttatcgccactggcagcagccactggtaacaggattagcagagcgaggtatgtaggcggtgctacagagttcttgaagtggtg
gcctaactacggctacactagaaggacagtatttggtatctgcgctctgctgaagccagttaccttcggaaaaagagttggtagctcttgatc
cggcaaacaaaccaccgctggtagcggtggtttttttgtttgcaagcagcagattacgcgcagaaaaaaaggatctcaagaagatcctttg

atcttttctacggggtctgacgctcagtggaacgaaaactcacgttaagggattttggtcatgagattatcaaaaaggatcttcacctagatcc
ttttaaattaaaaatgaagttttaaatcaatctaaagtatatatgagtaaacttggtctgacagttagaaaaactcatcgagcatcaaatgaaac
tgcaatttattcatatcaggattatcaataccatatttttgaaaaagccgtttctgtaatgaaggagaaaactcaccgaggcagttccataggat
ggcaagatcctggtatcggtctgcgattccgactcgtccaacatcaatacaacctattaatttcccctcgtcaaaaataaggttatcaagtgag
aaatcaccatgagtgacgactgaatccggtgagaatggcaaaagtttatgcatttctttccagacttgttcaacaggccagccattacgctcgt
catcaaaatcactcgcatcaaccaaaccgttattcattcgtgattgcgcctgagcgagacgaaatacgcgatcgctgttaaaaggacaattac
aaacaggaatcgaatgcaaccggcgcaggaacactgccagcgcatcaacaatattttcacctgaatcaggatattcttctaatacctggaat
gctgttttcccggggatcgcagtggtgagtaaccatgcatcatcaggagtacggataaaatgcttgatggtcggaagaggcataaattccg
tcagccagtttagtctgaccatctcatctgtaacatcattggcaacgctacctttgccatgtttcagaaacaactctggcgcatcgggcttcccat
acaatcgatagattgtcgcacctgattgcccgacattatcgcgagcccatttatacccatataaatcagcatccatgttggaatttaatcgcggc
ctagagcaagacgtttcccgttgaatatggctcataacaccccttgtattactgtttatgtaagcagacagttttattgttcatgaccaaaatccct
taacgtgagttttcgttccactgagcgtcagaccccgtagcacatttccccgaaaagtgccacctgacgtctaagaaaccattattatcatgac
attaacctataaaaataggcgtatcacgaggcagaatttcagataaaaaaaatccttagctttcgctaaggatgatttctg

plac_100:

gaattcgcggccgcttctagagctttaccagacatctcccccacaagaattggccctaccaattcttcgcttatctgacctctggttcacaattt
cccaattaaaactcacatcaatgttgacagctagctcagtcctagggattgtgctagctcattatccctacacaacacaattggcagtgccactt
ttacacaacgtgtgacaaggagatgagcaacagactcattacacgatgtgcgtggactcctactagagaaagaggagaaatactagatgc
gtaaaggagaagaacttttcactggagttgtcccaattcttgttgaattagatggtgatgttaatgggcacaaattttctgtcagtggagagg
gtgaaggtgatgcaacatacggaaaacttacccttaaatttatttgcactactggaaaactacctgttccatggccaacacttgtcactactttc
ggttatggtgttcaatgctttgcgagatacccagatcatatgaaacagcatgactttttcaagagtgccatgcccgaaggttatgtacaggaa
agaactatatttttcaaagatgacgggaactacaagacacgtgctgaagtcaagtttgaaggtgatacccttgttaatagaatcgagttaaaa
ggtattgattttaaagaagatggaaacattcttggacacaaattggaatacaactataactcacacaatgtatacatcatggcagacaaacaa
aagaatggaatcaaagttaacttcaaaattagacacaacattgaagatggaagcgttcaactagcagaccattatcaacaaaatactccaatt
ggcgatggccctgtccttttaccagacaaccattacctgtccacacaatctgccctttcgaaagatcccaacgaaaagagagaccacatggtc
cttcttgagtttgtaacagctgctgggattacacatggcatggatgaactatacaaataataatactagagccaggcatcaaataaaacgaaa
ggctcagtcgaaagactgggcctttcgttttatctgttgtttgtcggtgaacgctctctactagagtcacactggctcaccttcgggtgggcctt
tctgcgtttatatactagagctcgagttgacggctagctcagtcctaggtacagtgctagcggatccttactagagaaaggtggtgaatacta
gagatgattgttttacccagacgcctgtcagacgaggttgccgatcgtgtgcgggcgctgattgatgaaaaaaacctggaagcgggcatg
aagttgcccgctgagcgccaactggcgatgcaactcggcgtatcacgtaattcactgcgcgaggcgctggcaaaactggtgagtgaagg
cgtgctgctcagtcgacgcggcggcgggacgtttattcgctggcgtcatgacacatggtcggagcaaaacatcgtccagccgctaaaaac
actgatggccgatgatccggattacagtttcgatattctggaagcccgctacgccattgaagccagcaccgcatggcatgcggcaatgcgc
gccacacctggcgacaaagaaaagattcagctttgctttgaagcaacgctaagtgaagacccggatatcgcctcacaagcggacgttcgtt
ttcatctggcgattgccgaagcctcacataacatcgtgctgctgcaaaccatgcgcggtttcttcgatgtcctgcaatcctcagtgaagcatag
ccgtcagcggatgtatctggtgccaccggtttttcacaactgaccgaacaacatcaggctgtcattgacgccatttttgccggtgatgctgac
ggggcgcgtaaagcaatgatggcgcaccttagttttgttcacaccaccatgaaacgattcgatgaagatcaggctcgccacgcacggatta
cccgcctgcccggtgagcataatgagcattcgagggagaaaaacgcatgatactagagtcacactggctcaccttcgggtgggcctttctg
cgtttatatactagagagagaatataaaaagccagattattaatccggctttttattattttactagagtactagtagcggccgctgcaggcttc
ctcgctcactgactcgctgcgctcggtcgttcggctgcggcgagcggtatcagctcactcaaaggcggtaatacggttatccacagaatca
ggggataacgcaggaaagaacatgtgagcaaaaggccagcaaaaggccaggaaccgtaaaaaggccgcgttgctggcgtttttccata
ggctccgcccccctgacgagcatcacaaaaatcgacgctcaagtcagaggtggcgaaacccgacaggactataaagataccaggcgtttc
cccctggaagctccctcgtgcgctctcctgttccgaccctgccgcttaccggatacctgtccgcctttctcccttcgggaagcgtggcgctttct

catagctcacgctgtaggtatctcagttcggtgtaggtcgttcgctccaagctgggctgtgtgcacgaaccccccgttcagcccgaccgctg
cgccttatccggtaactatcgtcttgagtccaacccggtaagacacgacttatcgccactggcagcagccactggtaacaggattagcagag
cgaggtatgtaggcggtgctacagagttcttgaagtggtggcctaactacggctacactagaaggacagtatttggtatctgcgctctgctg
aagccagttaccttcggaaaaagagttggtagctcttgatccggcaaacaaaccaccgctggtagcggtggtttttttgtttgcaagcagca
gattacgcgcagaaaaaaaggatctcaagaagatcctttgatcttttctacggggtctgacgctcagtggaacgaaaactcacgttaaggg
attttggtcatgagattatcaaaaaggatcttcacctagatccttttaaattaaaaatgaagttttaaatcaatctaaagtatatatgagtaaactt
ggtctgacagttagaaaaactcatcgagcatcaaatgaaactgcaatttattcatatcaggattatcaataccatatttttgaaaaagccgtttct
gtaatgaaggagaaaactcaccgaggcagttccataggatggcaagatcctggtatcggtctgcgattccgactcgtccaacatcaataca
acctattaatttcccctcgtcaaaaataaggttatcaagtgagaaatcaccatgagtgacgactgaatccggtgagaatggcaaaagtttatg
catttctttccagacttgttcaacaggccagccattacgctcgtcatcaaaatcactcgcatcaaccaaaccgttattcattcgtgattgcgcctg
agcgagacgaaatacgcgatcgctgttaaaaggacaattacaaacaggaatcgaatgcaaccggcgcaggaacactgccagcgcatcaa
caatattttcacctgaatcaggatattcttctaatacctggaatgctgttttcccggggatcgcagtggtgagtaaccatgcatcatcaggagta
cggataaaatgcttgatggtcggaagaggcataaattccgtcagccagtttagtctgaccatctcatctgtaacatcattggcaacgctacctt
tgccatgtttcagaaacaactctggcgcatcgggcttcccatacaatcgatagattgtcgcacctgattgcccgacattatcgcgagcccattt
atacccatataaatcagcatccatgttggaatttaatcgcggcctagagcaagacgtttcccgttgaatatggctcataacaccccttgtattac
tgtttatgtaagcagacagttttattgttcatgaccaaaatcccttaacgtgagttttcgttccactgagcgtcagaccccgtagcacatttcccc
gaaaagtgccacctgacgtctaagaaaccattattatcatgacattaacctataaaaataggcgtatcacgaggcagaatttcagataaaaa
aatccttagctttcgctaaggatgatttctg

plac_118:

gaattcgcggccgcttctagagctttaccagacatctccccccacaagaattggccctaccaattcttcgcttatctgacctctggttcacaattt
cccaattaaaactcacatcaatgttgacagctagctcagtcctagggattgtgctagctcattatccctacacaacacaattggcagtgccactt
ttacacaacgtgtgacaaggagatgagcaacagactcattacacgatgtgcgtggactcctactagagaaagaggagaaatactagatgc
gtaaaggagaagaacttttcactggagttgtcccaattcttgttgaattagatggtgatgttaatgggcacaaattttctgtcagtggagagg
gtgaaggtgatgcaacatacggaaaacttacccttaaatttatttgcactactggaaaactacctgttccatggccaacacttgtcactactttc
ggttatggtgttcaatgctttgcgagatacccagatcatatgaaacagcatgactttttcaagagtgccatgcccgaaggttatgtacaggaa
agaactatatttttcaaagatgacgggaactacaagacacgtgctgaagtcaagtttgaaggtgatacccttgttaatagaatcgagttaaaa
ggtattgattttaaagaagatggaaacattcttggacacaaattggaatacaactataactcacacaatgtatacatcatggcagacaaacaa
aagaatggaatcaaagttaacttcaaaattagacacaacattgaagatggaagcgttcaactagcagaccattatcaacaaaatactccaatt
ggcgatggccctgtccttttaccagacaaccattacctgtccacacaatctgccctttcgaaagatcccaacgaaaagagagaccacatggtc
cttcttgagtttgtaacagctgctgggattacacatggcatggatgaactatacaaataataatactagagccaggcatcaaataaaacgaaa
ggctcagtcgaaagactgggcctttcgttttatctgttgtttgtcggtgaacgctctctactagagtcacactggctcaccttcgggtgggcctt
tctgcgtttatatactagagctcgagttgacggctagctcagtcctaggtacagtgctagcggatccttactagagaaaggtggtgaatacta
gagatgattgttttacccagacgcctgtcagacgaggttgccgatcgtgtgcgggcgctgattgatgaaaaaaaacctggaagcgggcatg
aagttgcccgctgagcgccaactggcgatgcaactcggcgtatcacgtaattcactgcgcgaggcgctggcaaaactggtgagtgaagg
cgtgctgctcagtcgacgcggcggcgggacgtttattcgctggcgtcatgacacatggtcggagcaaaacatcgtccagccgctaaaaac
actgatggccgatgatccggattacagtttcgatattctggaagcccgctacgccattgaagccagcaccgcatggcatgcggcaatgcgc
gccacacctggcgacaaagaaaagattcagctttgctttgaagcaacgctaagtgaagacccggatatcgcctcacaagcggacgttcgtt
ttcatctggcgattgccgaagcctcacataacatcgtgctgctgcaaaccatgcgcggtttcttcgatgtcctgcaatcctcagtgaagcatag
ccgtcagcggatgtatctggtgccaccggtttttttcacaactgaccgaacaacatcaggctgtcattgacgccatttttgccggtgatgctgac
ggggcgcgtaaagcaatgatggcgcaccttagttttgttcacaccaccatgaaacgattcgatgaagatcaggctcgccacgcacggatta
cccgcctgcccggtgagcataatgagcattcgagggagaaaaacgcatgatactagagtcacactggctcaccttcgggtgggcctttctg

cgtttatatactagagagagaatataaaaagccagattattaatccggctttttattattttactagagtactagtagcggccgctgcaggcttc
ctcgctcactgactcgctgcgctcggtcgttcggctgcggcgagcggtatcagctcactcaaaggcggtaatacggttatccacagaatca
ggggataacgcaggaaagaacatgtgagcaaaaggccagcaaaaggccaggaaccgtaaaaaggccgcgttgctggcgtttttccata
ggctccgcccccctgacgagcatcacaaaaatcgacgctcaagtcagaggtggcgaaacccgacaggactataaagataccaggcgtttc
cccctggaagctccctcgtgcgctctcctgttccgaccctgccgcttaccggatacctgtccgcctttctcccttcgggaagcgtggcgctttct
catagctcacgctgtaggtatctcagttcggtgtaggtcgttcgctccaagctgggctgtgtgcacgaaccccccgttcagcccgaccgctg
cgccttatccggtaactatcgtcttgagtccaacccggtaagacacgacttatcgccactggcagcagccactggtaacaggattagcagag
cgaggtatgtaggcggtgctacagagttcttgaagtggtggcctaactacggctacactagaaggacagtatttggtatctgcgctctgctg
aagccagttaccttcggaaaaagagttggtagctcttgatccggcaaacaaaccaccgctggtagcggtggtttttttgtttgcaagcagca
gattacgcgcagaaaaaaaggatctcaagaagatcctttgatcttttctacggggtctgacgctcagtggaacgaaaactcacgttaaggg
attttggtcatgagattatcaaaaaggatcttcacctagatccttttaaattaaaaatgaagttttaaatcaatctaaagtatatatgagtaaactt
ggtctgacagttagaaaaactcatcgagcatcaaatgaaactgcaatttattcatatcaggattatcaataccatatttttgaaaaagccgtttct
gtaatgaaggagaaaactcaccgaggcagttccataggatggcaagatcctggtatcggtctgcgattccgactcgtccaacatcaataca
acctattaatttcccctcgtcaaaaataaggttatcaagtgagaaatcaccatgagtgacgactgaatccggtgagaatggcaaaagtttatg
catttctttccagacttgttcaacaggccagccattacgctcgtcatcaaaatcactcgcatcaaccaaaccgttattcattcgtgattgcgcctg
agcgagacgaaatacgcgatcgctgttaaaaggacaattacaaacaggaatcgaatgcaaccggcgcaggaacactgccagcgcatcaa
caatattttcacctgaatcaggatattcttctaatacctggaatgctgttttcccggggatcgcagtggtgagtaaccatgcatcatcaggagta
cggataaaatgcttgatggtcggaagaggcataaattccgtcagccagtttagtctgaccatctcatctgtaacatcattggcaacgctacctt
tgccatgtttcagaaacaactctggcgcatcgggcttcccatacaatcgatagattgtcgcacctgattgcccgacattatcgcgagcccattt
atacccatataaatcagcatccatgttggaatttaatcgcggcctagagcaagacgtttcccgttgaatatggctcataacaccccttgtattac
tgtttatgtaagcagacagttttattgttcatgaccaaaatcccttaacgtgagttttcgttccactgagcgtcagaccccgtagcacatttcccc
gaaaagtgccacctgacgtctaagaaaccattattatcatgacattaacctataaaaataggcgtatcacgaggcagaatttcagataaaaaa
aatccttagctttcgctaaggatgatttctg

4. Additional references

1. Trantidou, T., Dekker, L., Polizzi, K., Ces, O. & Elani, Y. Functionalizing cell-mimetic giant vesicles with encapsulated bacterial biosensors. *Interface Focus* **8**, 20180024 (2018).

2. Goers, L. *et al.* Whole-cell Escherichia coli lactate biosensor for monitoring mammalian cell cultures during biopharmaceutical production. *Biotechnol. Bioeng.* **114**, 1290–1300 (2017).

3. Gåserød, O., Smidsrød, O. & Skjåk-Bræk, G. Microcapsules of alginate-chitosan - I. A quantitative study of the interaction between alginate and chitosan. *Biomaterials* **19**, 1815–1825 (1998).

4. Mao, A. S. *et al.* Programmable microencapsulation for enhanced mesenchymal stem cell persistence and immunomodulation. *PNAS* **116**, 15392–15397 (2019).
